# Alanine Derived from *Ruminococcus_E bovis* Alleviates Energy Metabolic Disorders during the Peripartum Period by Providing Glucogenic Precursors

**DOI:** 10.34133/research.0682

**Published:** 2025-04-25

**Authors:** Fanlin Kong, Shuo Wang, Yijia Zhang, Chen Li, Dongwen Dai, Yajing Wang, Zhijun Cao, Hongjian Yang, Shengli Li, Wei Wang

**Affiliations:** ^1^ State Key Laboratory of Animal Nutrition and Feeding, Department of Animal Nutrition and Feed Science, College of Animal Science and Technology, China Agricultural University, Beijing 100193, China.; ^2^ Laboratory of Animal Neurobiology, Department of Basic Veterinary Medicine, College of Veterinary Medicine, Nanjing Agricultural University, Nanjing 210095, China.; ^3^ Department of Animal Nutrition and Feed Science, College of Animal Science, Xinjiang Agricultural University, Urumqi 830052, China.; ^4^ Department of Animal Nutrition and Feed Science, College of Animal Science and Technology, Ningxia University, Yinchuan 750021, China.

## Abstract

Peripartum dairy cows commonly experience energy metabolism disorders, which lead to passive culling of postpartum cows and a decrease in milk quality. By using ketosis peripartum dairy cows as a model, this study aims to elucidate the metabolic mechanism of peripartum cows and provide a novel way for managing energy metabolic disorders. From a cohort of 211 cows, we integrated multi-omics data (metagenomics, metabolomics, and transcriptomics) to identify key microbes and then utilized an in vitro rumen fermentation simulation system and ketogenic hepatic cells to validate the potential mechanisms and the effects of postbiotics derived from key microbes. Postpartum cows with metabolic disorders compensate for glucose deficiency through mobilizing muscle proteins, which leads to marked decreases in milk protein content. Concurrently, these cows experience rumen microbiota disturbance, with marked decreases in the concentrations of volatile fatty acids and microbial protein, and the deficiency of alanine (Ala) in microbial protein is correlated with the metabolic disorder phenotype. Metagenomic binning and in vitro fermentation assays reveal that *Ruminococcus_E bovis* (MAG 189) is enriched in amino acid biosynthesis functions and responsible for Ala synthesis. Furthermore, transcriptomic and metabolomic analyses of the liver in metabolic disorder cows also show impaired amino acid metabolism. Supplementation with Ala can alleviate ketogenesis in liver cell models by activating the gluconeogenesis pathway. This study reveals that *Ruminococcus_E bovis* is associated with host energy metabolism homeostasis by supplying glucogenic precursors to the liver and suggests the use of Ala as a method for the treatment of energy metabolism disorders in peripartum cows.

## Introduction

Peripartum represents a pivotal stage in the mammalian life cycle. It not only heralds the arrival of a new life but also presents a unique window for discerning risks faced by both the fetus and the mother [1]. Regrettably, due to the intensive genetic selection aimed at enhancing productivity, which impairs the metabolic adaptability of dairy cows, 30% to 50% of cows are culled during this peripartum phase [2]. The elevated nutrient requirements for milk production trigger adipose tissue mobilization, insulin resistance, and immunosuppression [3–5]. Thus, elucidating the pathogenesis mechanisms of energy metabolism disorders is crucial for formulating precautionary strategies. Peripartum dairy cows serve as an ideal animal model for investigating energy metabolic disorders, which can be extrapolated to human studies. This is particularly significant considering the dearth of human peripartum research, mainly attributed to ethical and practical constraints associated with studying peripartum women. Firstly, insulin resistance occurs concomitantly in both humans and dairy cows during the peripartum period. In humans, insulin resistance typically manifests as peripartum diabetes mellitus [6,7]. In dairy cows, it ultimately results in the development of ketosis and fatty liver as the end-stage manifestations [4,5]. Moreover, dairy cows display an increased frequency of standing and lying, as well as a tendency to distance themselves from the herd [8], which bears resemblance to the manifestations of peripartum anxiety and depression in women [9]. When compared with specific-pathogen-free mice as an animal model, dairy cows, being monotocous and having a gestation period of approximately 280 d, similar to humans, give birth to either 1 or occasionally 2 calves [10]. The aforementioned studies highlight the similarities in the parturition process, physiological characteristics, and psychological aspects between peripartum humans and dairy cows. Consequently, the primary aims of this study are to delve into the mechanisms of energy metabolism disorders in peripartum dairy cows and propose preventive measures, with the aspiration of providing valuable insights for the study of metabolic disorders in peripartum humans.

In humans, a high concentration of ketone bodies in the blood is termed ketoacidosis. In the context of dairy cows, the same condition is referred to as ketosis [11,12]. An abnormally high level of β-hydroxybutyrate (BHBA), one of the ketone bodies, in the blood (1.2 mmol/l) is considered a marker of metabolic disorders. A high BHBA concentration is believed to contribute to insulin resistance [5], immunosuppression [13], and damage to liver and mammary gland function via oxidative-stress-mediated apoptosis [14]. Generally, the fatty acids in the systemic circulation are used for milk fat synthesis and hepatic oxidation. However, an excessive amount of fatty acids invariably exceeds the liver’s metabolism capacity [15]. As a result, the acetyl coenzyme A from fatty acid oxidation utilizes a limited amount of oxaloacetic acid in the tricarboxylic acid cycle and has to produce excess ketone bodies [16]. Unfortunately, the etiology of ketosis remains unknown. Hence, it is essential to clarify the pathogenesis mechanism of energy metabolism disorders to establish precautionary measures.

Volatile fatty acids (VFAs) are mainly produced from plant fibers by rumen microbial fermentation and make up 70% of the host’s energy source [17]. Primary studies have found that individual VFA concentrations were changed in ketosis cows [18,19], suggesting that rumen dysfunction may play an important role in the development of ketosis. Additionally, microbial protein (MCP) is the end product of feed protein by rumen microbial fermentation and makes up 50% of the protein source of the host [20]. The development of ketosis may also depend on protein metabolism, as approximately 20% of glucose comes from glucogenic amino acids. Studies also revealed that most amino acids are decreased in the blood [21–23] and milk [23] of ketosis cows. Investigations about amino acid functions indicate that essential amino acids except for Lys and Leu are the glucogenic amino acids for dairy cows [24]. The significance of the gut–liver axis in shaping intestinal amino acid profiles has been emphasized in human studies [25]. For peripartum women, a nested case–control study also revealed that the amino acid metabolism of peripartum women was up-regulated [26] and branched amino acids may be considered a biomarker of gestational diabetes mellitus [27]. Concerning the rapidly increasing glucose demand for lactation, it has been hypothesized that the glucose supply is supported by increased utilization of glucogenic amino acids for liver gluconeogenesis. We hypothesized that the rumen microbiota is responsible for the development of ketosis by decreasing amino acid supplementation for energy metabolism and that the absence of key microbes makes it difficult to alleviate ketosis via manipulating the rumen microbiota.

Cows are a well-established animal model for exploring the role of gastrointestinal microbiota in lactating mammals’ diseases [28,29]. In this nested case–control study, we applied metagenome assembly and binning strategies to reconstruct microbial population genomes from the microbiota samples of ketosis cows and analyzed the gene expression and metabolite concentration in the liver using RNA sequencing (RNA-seq) and metabolomics. After filtering the potential microbes associated with ketosis development, we used a self-developed in vitro rumen fermentation simulation system to evaluate the effects of the key microbes’ addition. Next, we verified the causality of the addition of key metabolites produced by microbes in alleviating ketogenesis, as the unclear causality between microbial metabolites and host metabolism represents a significant bottleneck restricting current achievements in microbial research [30,31]. Our results demonstrate an association between the rumen–liver axis and energy metabolism in dairy cows during the peripartum period at an unprecedentedly high level of taxonomic resolution. This study will primarily contribute to the healthy development of dairy cattle farming by clarifying the pathogenesis of ketosis and providing potential therapeutic approaches. Secondarily, it will provide a mechanistic reference for energy metabolism disorders during the peripartum period of mammals.

## Results

### The liver glycogen of ketosis cows was exhausted and mobilized more body protein and fat

**Fig. 1.**
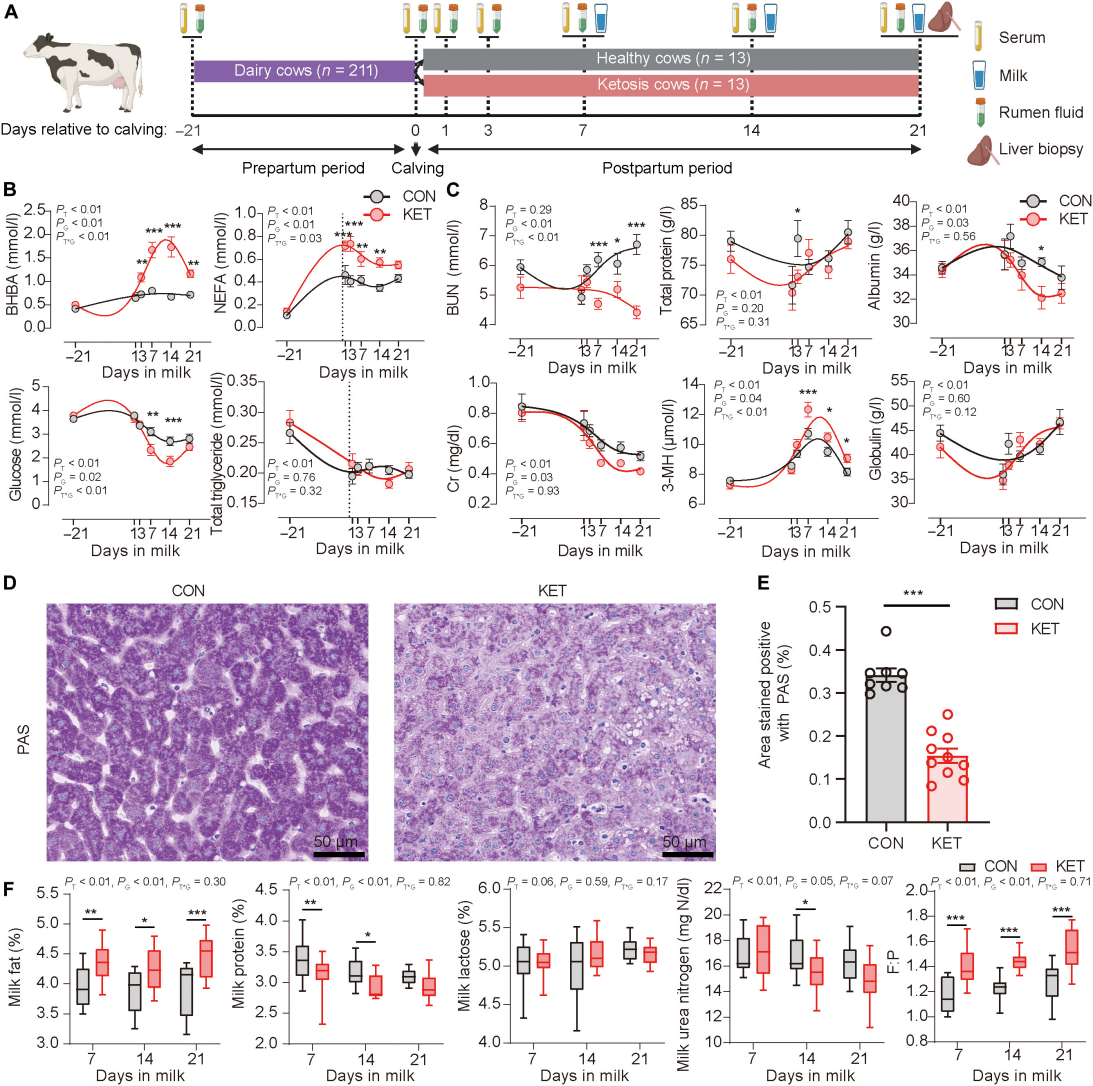
Changes in milk composition, serum nitrogen, and serum energy metabolism indices during the peripartum period and liver glycogen as demonstrated by periodic acid–Schiff (PAS) on day 21 in healthy (CON) and ketosis (KET) cows. (A) Schematic of the nested case–control study. (B) Longitudinal change in serum energy metabolism indices. The lowness function in the GraphPad Prism (v 9.3) was used to create a smooth line. (C) Longitudinal change in serum nitrogen metabolism indices. (D) Representative images of PAS staining of liver sections. (E) Morphometric analysis of the area positively stained for PAS in the liver of cows in 2 groups. Analysis was performed using Image-Pro Plus by measuring the total area stained as a percentage of the total area per digital photo. (F) Longitudinal change in milk composition and ratio of milk fat to milk protein (F:P). The boxplots show the mean (centerline), quartiles (box limits), and max to min range (whiskers). Dots correspond to individual samples. *P* values for blood indices and milk compositions were calculated using the mixed model. The liver index was calculated using unpaired 2-tailed *t* tests. *P < 0.05; **P < 0.01; ***P < 0.001. *N* = 13. BHBA, β-hydroxybutyrate; NEFA, nonesterified fatty acid; BUN, blood urea nitrogen; Cr, creatinine; 3-MH, 3-methylhistidine.

For the nested case–control study (Fig. 1A), we first detected the energy and nitrogen metabolic indices. There were interaction effects between time and group on BHBA, nonesterified fatty acid (NEFA), and glucose concentrations (Fig. 1B). The NEFA concentrations were higher in the ketosis cows (KET) on days 1, 3, 7, and 14 when compared with those in the healthy cows (CON), while glucose concentrations were lower in the KET group on days 7 and 14 (Fig. 1B). The BHBA concentrations were higher on days 3, 7, 14, and 21 (Fig. 1B).

The blood urea nitrogen (BUN) and 3-methylhistidine (3-MH) concentrations were affected by the interaction between time and group (Fig. 1C). After calving, the BUN concentrations were higher and 3-MH concentrations were lower in CON on days 7, 14, and 21 when compared with those in the KET group (Fig. 1C). Only time effects were significant on total protein and globulin concentrations (Fig. 1C). Although the creatinine (Cr) concentrations were not affected by the interaction between time and group, the group effect was significant and the average Cr concentration in the CON group was higher than that in the KET group (Fig. 1C). Liver periodic acid–Schiff (PAS) staining (Fig. 1D and E) showed that the glycogen area was lower in the KET group on day 21.

The milk content is the outcome of body energy and nitrogen metabolism. All of the milk compositions were affected by the group instead of the interaction between time and group (Fig. 1F). The milk fat content was higher and the milk protein content was lower in the KET group than in the CON group (Fig. 1F). The ratio of milk fat to milk protein (F:P) was higher in the KET group (Fig. 1F).

### The amino acid metabolism of rumen microbiota was weakened in the ketosis cows, and it was attributed to the lack of 2 clusters of the metagenome-assembled genome

Next, we compared the function profiles of the rumen microbiome between ketosis cows and healthy cows. For metagenomics sequencing, the composition of rumen microbiota in the KET group was significantly different from it in the CON group on day 3 (Fig. S1A to D). After filtering the unchanged pathways, the abundance of multiple pathways involved in amino acid metabolism was lower in the KET group (Fig. S2A and B).

After functional comparisons, we further found that the key microbes were responsible for functional changes. We assembled a metagenome-assembled genome (MAG), and a total of 293 high-quality MAGs were obtained from all samples (mean completeness = 89.18%, mean contamination = 3.87%, and mean N50 = 16.1 kilobases; Fig. S3). After that, 293 MAGs were dereplicated at an average nucleotide identity threshold of 99%, resulting in a final set of 190 nonredundant MAGs with strain-level resolution (Table S1). The numbers of MAGs and nonredundant MAGs were higher in the KET group than in the CON group on all days (Fig. S4A and B). The composition of MAG in the KET group was also different from that in the CON group on day 3 (Fig. S4C and D). The MAG proportions of 4.7% and 95.3% were assigned to archaea and bacteria (Fig. S4E and Table S2), respectively. *Methanobrevibacter* was the dominant species of archaea (77.8%), and *Ruminococcus_E* was the dominant bacterial species (10.5%). The average number and proportion of genes related to metabolism were higher in the CON group on all days (Fig. S5A). Amino acid metabolism, biosynthesis of other secondary metabolites, energy metabolism, glycan biosynthesis and metabolism, metabolism of cofactors and vitamins, metabolism of other amino acids, and nucleotide metabolism pathways were lower in the KET group than in the CON group on day 3 (Fig. S5B).

**Fig. 2.**
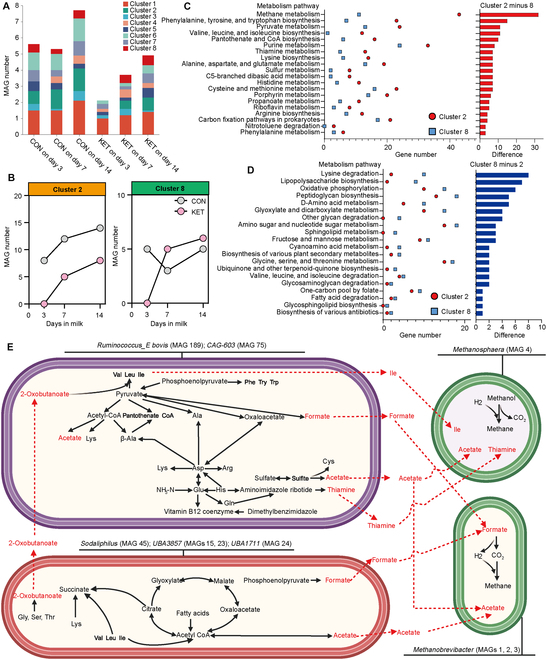
Clustering analysis of the Kyoto Encyclopedia of Genes and Genomes (KEGG) level 3 pathway composition was used to find the key ruminal metagenome-assembled genomes (MAGs) and their potential functions that may be responsible for ketosis. (A) Cluster composition of MAGs in the healthy (CON) and ketosis (KET) cows on days 3, 7, and 14. (B) Changes in MAG number in clusters 2 and 8. (C) The dominant metabolism functions of KEGG level 3 in cluster 2 when compared with those in cluster 8. The dot plot shows the dominant functions of cluster 2, which means that the average gene numbers per MAG in each metabolism pathway were higher in cluster 2. The top 20 pathways are presented. The bar plot shows the difference in gene numbers of individual pathways. (D) The dominant metabolism functions of KEGG level 3 in cluster 8 when compared with those in cluster 2. The dot plot shows the dominant functions of cluster 8, which means that the average gene numbers per MAG in each metabolism pathway were higher in cluster 8. The top 20 pathways are presented. The bar plot shows the difference in gene numbers of individual pathways. (E) Probable metabolic interactions between MAGs in clusters 2 and 8. Both clusters 2 and 8 were absent in the KET group on day 3. CoA, coenzyme A.

To filter the key species and clarify the pathogenesis mechanism, we further grouped MAGs by gene number in Kyoto Encyclopedia of Genes and Genomes level 3 pathways into 8 clusters (Fig. S6). Interestingly, the MAGs in both clusters 2 and 8 were absent in the KET group on day 3 (Fig. 2A). Longitudinal changes showed that the MAG numbers in cluster 2 increased gradually after calving in the CON group, and the MAG number in cluster 8 was stable (Fig. 2B). In the KET group, both these parameters increased after calving (Fig. 2B). The above evidence suggested the potential complementation of metabolism between clusters 2 and 8. Figure 2C shows that the dominant metabolic functions of MAGs in cluster 2 were methane metabolism and most amino acid metabolism biosynthesis pathways, such as Phe, Tyr, Trp, Val, Leu, Ile, Lys, Ala, Asp, and Glu metabolism. Pyruvate metabolism, which is the critical energy metabolism pathway of rumen microbiota, was also enriched in cluster 2 (Fig. 2C). Several glycan biosynthesis and metabolism pathways were enriched in cluster 8 (Fig. 2D).

According to the results shown in Fig. 2C and D, we identified the probable metabolic interactions between MAGs in clusters 2 and 8 (Fig. 2E). MAGs 1 to 4 in cluster 2 are archaea and good at methane production. Hence, we divided cluster 2 into 2 parts, which contained bacteria including *Ruminococcus_E bovis* (MAG 189) and *CAG-603* (MAG 75) and archaea *Methanobrevibacter* (MAG 1 to 3) and *Methanosphaera* (MAG 4). Based on the dominant pathways in each cluster in Fig. 2C and D, *Ruminococcus_E bovis* (MAG 189) and *CAG-603* (MAG 75) provided Ile, acetate, and thiamine for *Methanosphaera* (MAG 4) and formate and acetate for *Methanobrevibacter* (MAG 1 to 3). 2-Oxobutanoate, which is a product of Gly, Ser, and Thr catabolism, might be provided by *Sodaliphilus* (MAG 45), *UBA3857* (MAGs 15 and 23), and *UBA1711* (MAG 24) for *Ruminococcus_E bovis* (MAG 189) and *CAG-603* (MAG 75).

### MCP production derived from rumen microbiota decreased, and Ala within it was associated with the host’s energy metabolic indices

**Fig. 3.**
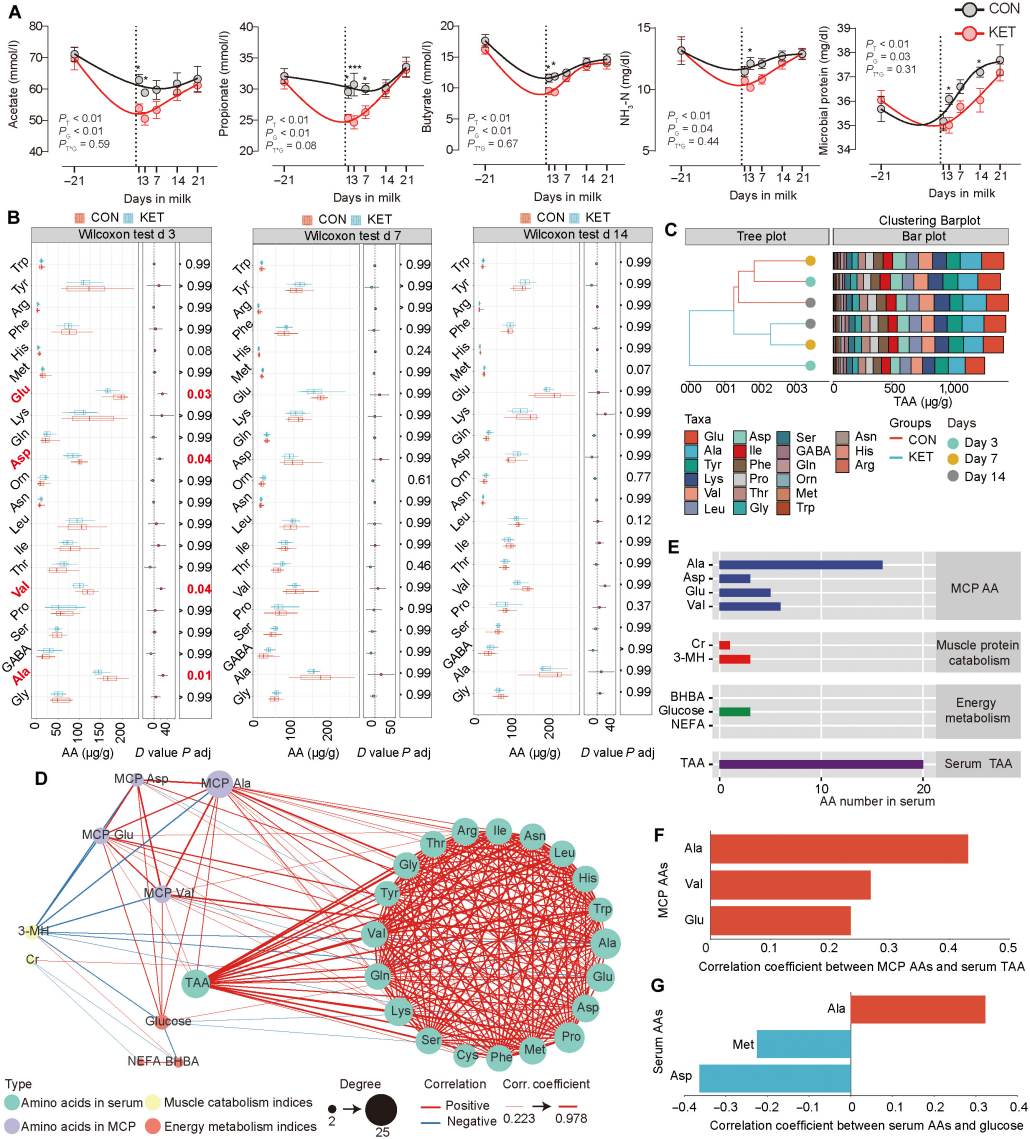
Energy and nitrogen supplementation from the rumen microbiota to the host was changed in ketosis (KET) cows when compared with healthy (CON) cows. (A) Volatile fatty acids (VFAs), NH_3_-N, and microbial protein (MCP) concentrations. The lowness function in the GraphPad Prism (v 9.3) was used to create a smooth line. Data are presented as mean ± standard error of the mean (SEM). *P* values for blood indices and milk compositions were calculated using the mixed model. *P < 0.05; **P < 0.01; ***P < 0.001. (B) Differences in amino acid concentrations in MCP between the CON and KET groups on different days. *P* values were calculated using a 2-tailed Wilcoxon rank test combined with the Bonferroni–Dunn method. (C) Amino acid composition of MCP on days 3, 7, and 14. The clustering tree was generated via Bray–Curtis. (D) Spearman’s correlation was conducted, and the significant correlations (*P* < 0.05) are presented. The nodes with different colors indicate the index types. The size of the node indicates the degree of the node. The red and blue lines between 2 nodes indicate positive and negative correlations, respectively. The thickness of the line indicates the absolute value of the correlation coefficient. (E) The number of significant correlations between amino acid concentrations in MCP, muscle protein catabolism indices, energy metabolism indices, total amino acids (TAA), and amino acid concentrations in serum. (F) Correlation coefficient between MCP amino acid concentrations and serum TAA concentration. (G) Correlation coefficient between serum amino acid concentrations and serum glucose concentration. *N* = 13. GABA, γ-aminobutyric acid; AA, amino acid; *P* adj, adjusted *P*.

Rumen microbiota is crucial for the conversion of dietary carbohydrates and nitrogen to VFAs and MCP, and the above MAG analyses revealed the potential changes of these conversions. Thus, Fig. 3A shows the longitudinal changes in individual VFAs, ammonia (NH_3_-N), and MCP concentrations. All parameters were affected by the time and group effects instead of the interaction between time and group (Fig. 3A). The averages of individual VFAs, NH_3_-N, and MCP concentrations were higher in the CON group when compared with those in the KET group (Fig. 3A). The amino acid metabolism was changed according to the MAG results. Hence, we further analyzed the amino acid composition of the MCP (Fig. 3B and C). The Glu, Asp, Val, and Ala concentrations were significantly lower on day 3 in the KET group (Fig. 3B). On days 7 and 14, the amino acid concentration did not change significantly (Fig. 3B). Moreover, the clustering tree showed that the amino acid composition of the KET group on day 3 was different from that of the CON group (Fig. 3C).

Figure 3D shows that the Asp, Glu, Val, and Ala concentrations in the MCP were significantly correlated with serum amino acid concentrations, with Ala having the highest number of positive correlations (16) with serum amino acid concentrations (Fig. 3E). The 3-MH concentration was negatively correlated with Asp, Glu, and Val concentrations in the MCP, serum glucose, and Ala (Fig. 3D). NEFA concentration was positively correlated with BHBA concentration, and BHBA concentration was negatively correlated with glucose concentration (Fig. 3D). Furthermore, Ala in MCP was correlated with serum total amino acids (TAA), with the highest correlation coefficient when compared to other amino acids of MCP (Ala, 0.43; Val, 0.27; Glu, 0.23) (Fig. 3F). The concentration of Ala, the only amino acid in MCP, was positively correlated with serum glucose concentration (Fig. 3G).

### Supplementation of *Ruminococcus bovis* JE7A12 to an in vitro rumen fermentation simulation system increased MCP and Ala production

**Fig. 4.**
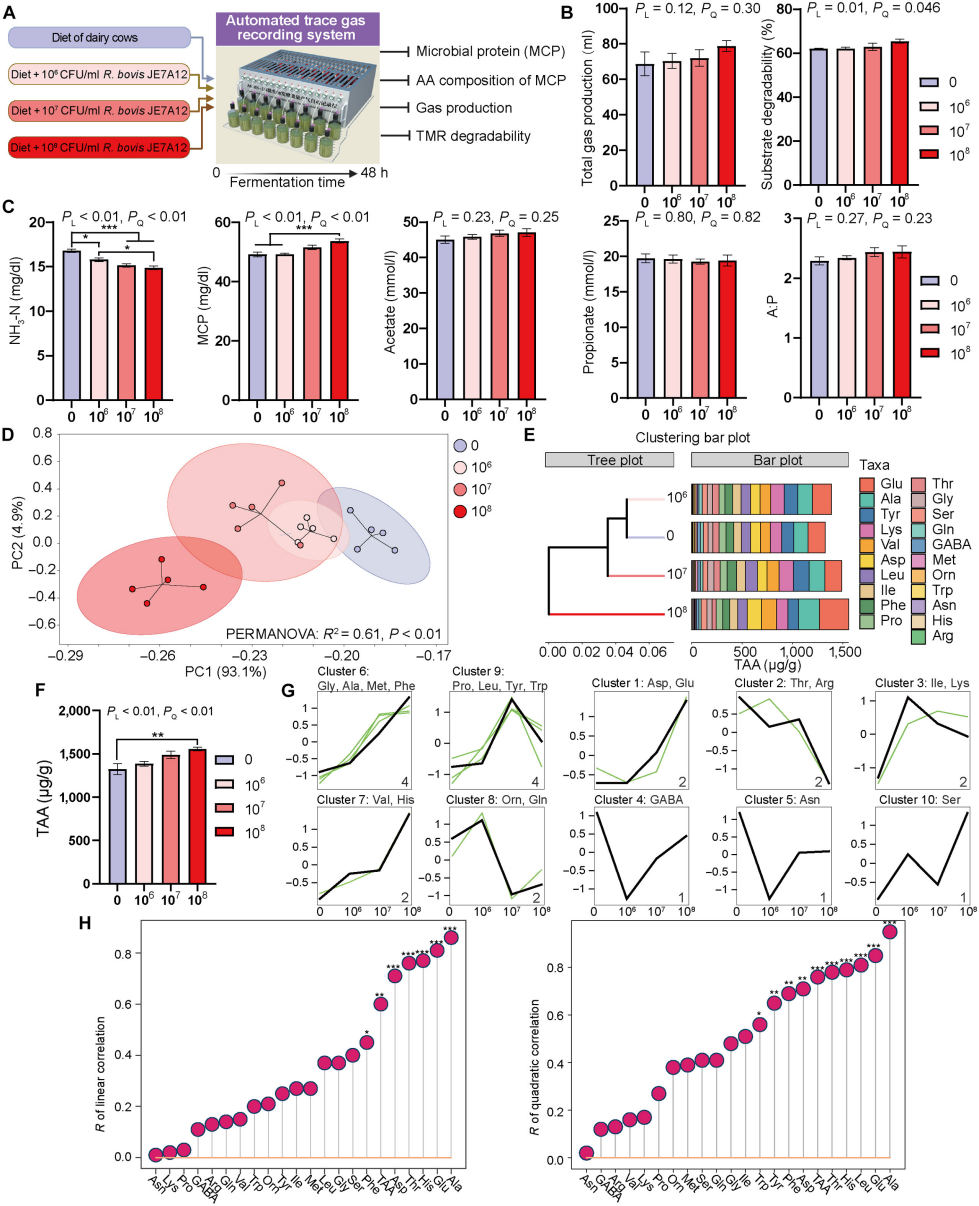
The amino acid composition of the MCP is changed by *Ruminococcus bovis* JE7A12 supplementation via in vitro fermentation. (A) Schematic of an in vitro fermentation experiment. The dairy cow diet was used as the substrate, and different amounts of *R. bovis* JE7A12 (10^6^, 10^7^, and 10^8^ CFU/ml) were added into the fermentation system for 48 h. (B) Total gas production and substrate degradability at 48 h. (C) Fermentation parameters of fermentation broth. (D) Principal component analysis (PCA) plot of amino acid composition in MCP. Permutational multivariate analysis of variance (PERMANOVA) was used to examine the distribution, and *P* < 0.05 was considered a significant distribution. (E) Amino acid composition of MCP. The clustering tree was generated via Bray–Curtis. (F) TAA concentration in the microbiota of fermentation fluid. (G) Mfuzz clustering of the changing trend of amino acid concentration among different groups. (H) Rank of Pearson’s *R* correlation coefficient of linear and quadratic correlation between amino acid concentrations and amounts of *R. bovis* JE7A12. Statistical significance was calculated using one-way analysis of variance (ANOVA) with Tukey’s multiple comparisons. *P < 0.05; **P < 0.01; ***P < 0.001. Linear and quadratic relationships were examined, and *P* values are presented as *P*_L_ and *P*_Q_. *N* = 5. TMR, total mixed ration; A:P, acetate:propionate.

After identifying the key microbes and potential mechanism for ketosis development, we examined the effects of different amounts of *Ruminococcus bovis* JE7A12 supplementation on the fermentation parameters and amino acid composition of MCP to validate the key role of *R. bovis* JE7A12 in rumen amino acid metabolism (Fig. 4A). Supplementation of this strain did not affect gas production (Fig. 4B and Fig. S7A) and increased substrate degradability and time to reach half the ideal maximum gas production by linear and quadratic ways (Fig. 4B and Fig. S7B). For fermentation parameters, supplementation linearly and quadratically decreased pH (Fig. S7C) and NH_3_-N concentration (Fig. 4C) and increased MCP concentration of fermentation fluid with no effect on VFA concentrations (Fig. 4C). Principal component analysis plot combined with permutational multivariate analysis of variance shows the distinct distribution of the amino acid composition of MCP from different groups (Fig. 4D). The concentrations of Glu, Ala, Tyr, Lys, and Val were the top 5 amino acids in the MCP (Fig. 4E), and the TAA concentrations were increased with *R. bovis* JE7A12 supplementation in linear and quadratic ways (Fig. 4F). Mfuzz clustering found that Gly, Ala, Met, and Phe concentrations were changed with that same trend among different groups and increased with supplementation visually (Fig. 4G). Finally, the rank of *R* coefficient from linear and quadratic correlations in Fig. 6H shows that Ala, Glu, His, Thr, Asp, TAA, and Phe concentrations were positively correlated with the supplementation amount of *R. bovis* JE7A12 and Ala, Glu, Leu, His, Thr, TAA, Asp, Phe, Tyr, and Trp concentrations were quadratically changed with the supplementation amount of *R. bovis* JE7A12.

### Hepatic gene expression and metabolite concentration revealed that amino acid metabolism was depressed in ketosis cows

**Fig. 5.**
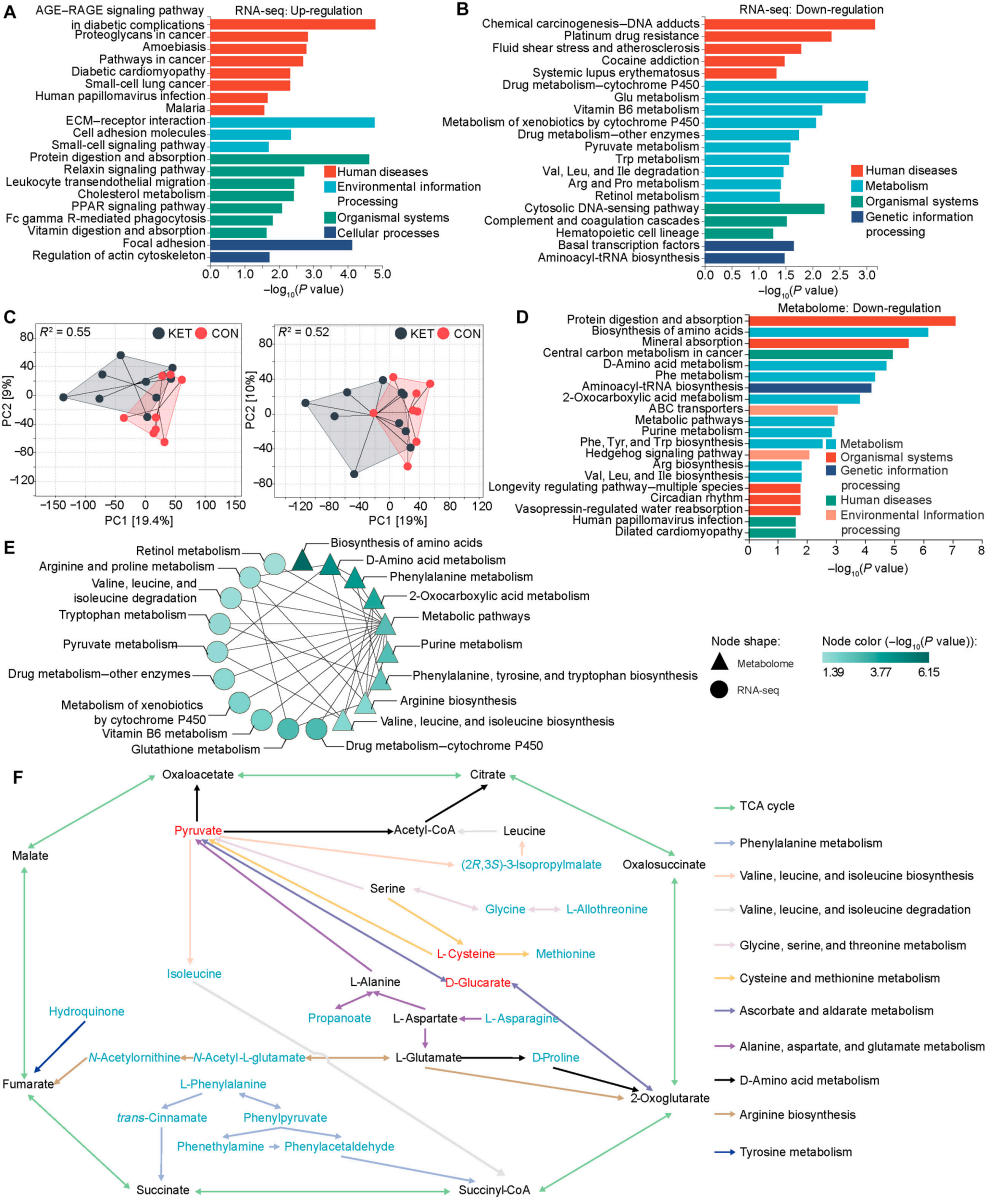
Hepatic amino acid metabolism was disturbed on day 21 in ketosis (KET) cows when compared with that in healthy (CON) cows. (A and B) The up- or down-regulating pathways of hepatic genes. The top 20 pathways are presented. (C) PCA based on *Z*-score transformation was used to visualize the metabolome (left: positive mode; right: negative mode). (D) The down-regulating pathways of hepatic metabolites. The top 20 pathways are presented. (E) The relationship of significant down-regulation pathways from metabolomics and transcriptomics. The upstream and downstream pathways are connected by a line. (F) Visualization of the regulation network about significantly different metabolites. The red and blue words indicate the up- or down-regulating metabolites in the KET group when compared with those in the CON group, respectively. The black words indicate unchanged metabolites. The arrows with diverse colors indicate the metabolism pathways. *N* = 10. AGE–RAGE, advanced glycation end products–receptor for advanced glycation end products; ECM, extracellular matrix; PI3K, phosphoinositide 3-kinase; PPAR, peroxisome proliferator-activated receptor; RNA-seq, RNA sequencing; tRNA, transfer RNA; ABC, ATP synthase (ATP)-binding cassette; TCA, tricarboxylic acid.

Although we clarified the mechanism of the rumen microbiome and the potential key metabolite (Ala), the responses of host metabolism were still not clear. We detected liver metabolism and gene expression on the last day of the experiment via biopsy. Figure 5A and B show the top 20 up-regulated and down-regulated pathways of hepatic genes, respectively. Most of the up-regulated pathways were involved in human diseases and organismal systems without metabolism pathways (Fig. 5A). Conversely, many metabolism pathways, including drug metabolism—cytochrome P450, glutathione, vitamin B6, pyruvate, and many amino acid metabolism pathways were significantly down-regulated in the KET group compared to those in the CON group (Fig. 5B). For metabolomics, a principal component analysis plot showed a clear distinction between the 2 groups in positive or negative mode (Fig. 5C). There were no significantly up-regulated pathways. The biosynthesis of amino acids, d-amino acid metabolism, and several amino acid metabolism pathways (Phe, Tyr, Trp, Arg, Val, Leu, and Ile) were down-regulated. There were upstream and downstream relationships among the significantly downregulated pathways derived from metabolomics and transcriptomics (Fig. 5E). Most of the amino acids and related metabolites were down-regulated in the liver, including Gly, Met, Asp, Ile, Pro, and Phe (Fig. 5F).

### Ala supplementation for ketogenic hepatic cells enhanced gluconeogenesis and alleviated ketogenesis

**Fig. 6.**
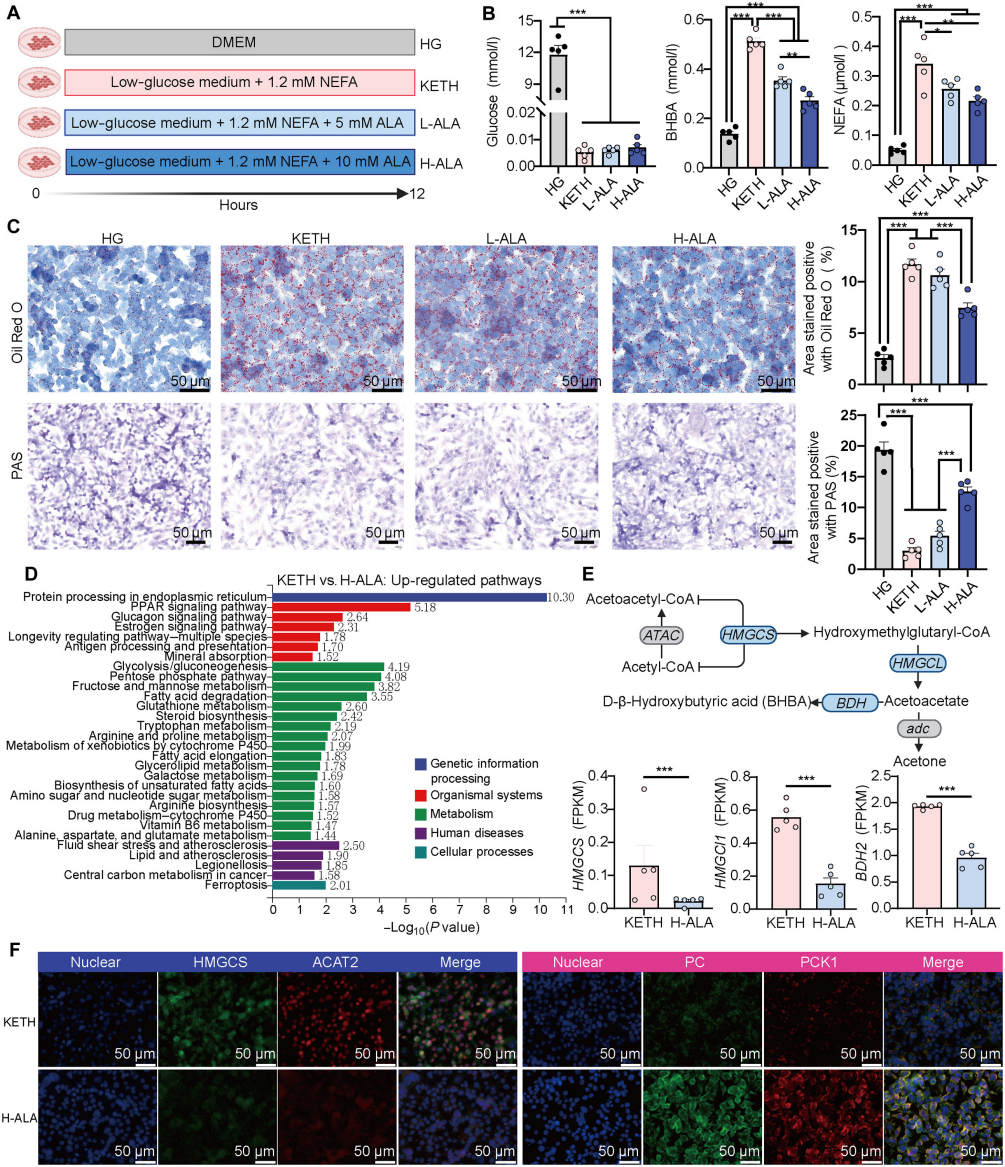
Ala supplementation for ketogenic hepatic cells enhanced gluconeogenesis and alleviated ketogenesis. (A) Schematic of the hepatic cell experiment. Hepatic cells in the high-glucose (HG) group were maintained in Dulbecco’s modified Eagle’s medium (DMEM)/Gibco (4.5 g/l). Hepatic cells in the ketogenic hepatocyte (KETH) group were maintained in DMEM/low glucose (1 g/l) and supplied with 1.2 mM NEFA for 12 h. Hepatic cells in low-Ala supplementation (L-ALA) and high-Ala supplementation (H-ALA) groups were also maintained in DMEM/low glucose with 1.2 mM NEFA and then supplied with 5 or 10 mM Ala for 12 h. (B) Concentrations of glucose, BHBA, and NEFA in the supernatant of media. Statistical significance was calculated using one-way ANOVA with Tukey’s multiple comparisons. *P < 0.05; **P < 0.01; ***P < 0.001. (C) Representative images of Oil Red O and PAS staining. Analysis was performed using Image-Pro Plus by measuring the total area stained as the percentage of the total area per digital photo. Data are presented as mean ± SEM. Statistical significance was calculated using one-way ANOVA with Tukey’s multiple comparisons. *P < 0.05; **P < 0.01; ***P < 0.001. (D) Significantly up-regulated pathways of differentially expressed genes in the H-ALA group compared with those of the KETH group. The top 30 *P* values of pathways are presented. (E) Expression of genes in the ketone body biosynthesis pathway. The gray ellipse indicates the undetected genes. The blue ellipse indicates the down-regulated genes in the H-ALA group. Statistical significance was calculated using DESeq2 with Benjamini–Hochberg (BH) multiple comparisons. *P < 0.05; **P < 0.01; ***P < 0.001. (F) Immunofluorescence for ketogenic, hydroxymethylglutaryl-coenzyme A synthase (HMGCS) and acetyl coenzyme A acetyltransferase 2 (ACAT2), and glucogenic proteins, pyruvate carboxylase (PC) and phosphoenolpyruvate carboxykinase 1 (PCK1), for the KET and H-ALA groups. The nuclear dye 4′,6-diamidino-2-phenylindole (DAPI; blue) was used. *N* = 5. FPKM, fragments per kilobase of transcript per million mapped reads.

We found that Ala in MCP may be responsible for host amino acid metabolism and gluconeogenesis (Fig. 3). Therefore, we further established a ketogenic hepatic cell model and investigated the effects of Ala supplementation on gluconeogenesis and ketogenesis (Fig. 6A). First, the replacement of energy sources from glucose to NEFA in the ketogenic hepatocyte (KETH) group enhanced BHBA concentration (Fig. 6B) and fat deposition and reduced glycogen storage (Fig. 6C) when compared to those in the high-glucose (HG) group. The low-Ala supplementation (L-ALA) group reduced NEFA and BHBA concentrations (Fig. 6B) and did not affect fat deposition and glycogen storage compared with those in the KETH group (Fig. 6C). The high-Ala supplementation (H-ALA) group reduced BHBA concentration (Fig. 6B) and fat deposition and enhanced glycogen storage (Fig. 6C) compared to those in the KETH and L-ALA groups.

RNA-seq showed that the gene expression pattern in the H-ALA group was different from that in the KETH group (Fig. S8A). A total of 960 genes were up-regulated and 630 genes were down-regulated (Fig. S8B). Only propanoate metabolism was down-regulated in the H-ALA group when compared to that in the KETH group (Fig. S8C). The glycolysis/gluconeogenesis and Ala metabolism pathways were up-regulated in the H-ALA group (Fig. 6D). The expression of key genes involved in ketone body biosynthesis, including *HMGCS*, *HMGCL1*, and *BDH2*, were down-regulated in the H-ALA group when compared to that in the KETH group (Fig. 6E).

Immunofluorescence confirmed the down-regulation of key enzymes of ketogenesis including hydroxymethylglutaryl-coenzyme A synthase and acetyl coenzyme A acetyltransferase 2 (Fig. 6F and Fig. S9A and C) and the up-regulation of key enzymes of gluconeogenesis including pyruvate carboxylase in the H-ALA group when compared with those of other groups (Fig. 6F and Fig. S9B and D). The mean fluorescence intensity (MFI) of phosphoenolpyruvate carboxykinase 1 was higher in the KETH group when compared with those in other groups and was also higher in the L-ALA and H-ALA groups than in the HG group (Fig. 6F and Fig. S9B and D). The MFI of peroxisome proliferator-activated receptor α was higher in the H-ALA group when compared with those in other groups (Fig. S9E and F). The MFI of diacylglycerol *O*-acyltransferase 2 was higher in the KETH and L-ALA groups when compared with that in the HG group. It was lower in the H-ALA group when compared with that in the KETH group (Fig. S9E and F).

## Discussion

**Fig. 7.**
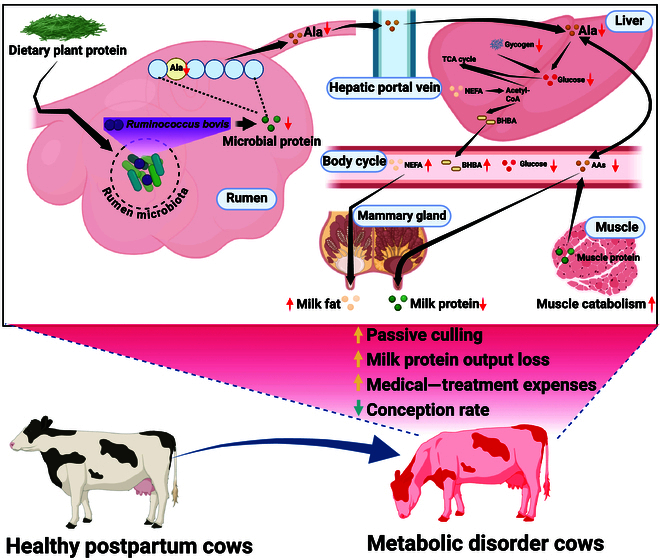
The pathogenesis of ketosis in dairy cows induced by Ala deficiency. Upon the intake of dietary plant protein, the rumen microbiota is responsible for its conversion into and supply of MCP to the host. However, a deficiency of ruminal *R. bovis* results in a decrease in the proportion of Ala within MCP. Despite an increase in muscle protein breakdown at this stage to offset the amino acid deficiency, the lack of Ala leads to a scarcity of precursors in the liver for gluconeogenesis. This, in turn, compels the conversion of NEFA into BHBA. Consequently, the concentration of BHBA in the blood rises, while that of glucose declines. In milk, the concentration of milk fat increases, whereas the concentration of milk protein decreases.

Herein, we analyzed the longitudinal changes in energy and nitrogen metabolism indices. We found that glucose deficiency may be responsible for ketosis. For ketosis cows, higher NEFA concentrations indicated greater modulization of body fat, which may provide more energy to compensate for the energy from glucose. Liver glycogen staining also supported the observed glucose deficiency and increased glycogen consumption in ketosis cows. 3-MH and Cr are correlated with the skeletal muscle mass. Although our results showed that ketosis cows mobilized more muscle protein than healthy cows, the milk protein composition was still lower in ketosis cows than in healthy cows (Fig. 7). Several studies have measured the milk composition of ketosis cows [23], which is consistent with our results. The contradictory results indicated that muscle protein is used as an energy source instead of milk protein synthesis. A high percentage of mothers from low- and middle-income countries were malnourished [32] and may encounter problems similar to those experienced by postpartum dairy cows. The energy and nitrogen negative balance is dependent on the intake (dry matter intake) and output (milk production, maintenance, and heat increment). Hence, further study is needed to quantify the energy profile of ketosis cows.

More than 70% of the energy requirements [20] and 50% of the nitrogen requirements of cows [17] are met by VFAs and MCP, which are produced by the rumen microbiota [33]. Metagenomic analyses revealed that the rumen microbiota of ketosis cows was significantly different from that of healthy cows. A cross-sectional study using metagenomics also identified the differences between ketosis and healthy cows [18]. Importantly, our study found that the differences in the rumen microbiota of ketosis cows occurred on days 3 and 7, which was earlier than the peak of the BHBA curve. This chronological order provides us with a causal relationship between ketosis and rumen microbiota instead of a correlation relationship.

In our study, the higher number of species indicated that the rumen microbiota became more complex and competitive in ketosis cows and had decreased specificity to ferment the diet to support the host energy requirements according to their co-occurrence, which was consistent with a previous study [18]. Lower VFAs and MCP concentrations are the results of less microbiota specificity. Then, it could be due to an effect of the higher rate of microbial turnover in the rumen of ketosis cows, as shown by the enrichment of genetic information processing in ketosis cows on day 3. Hence, our results improve our understanding of the impact of rumen disruption not only on carbohydrate metabolism but also on nitrogen metabolism in ketosis cows.

Several metabolic pathways of rumen MAGs in ketosis cows were down-regulated after calving, particularly on day 3. *Sodaliphilus* was first described within the pig microbiome [34]. In our study, the lack of simultaneously present MAGs (day 3) demonstrated that synergistic interactions exist between the bacteria and archaea. Bioinformatic analysis revealed that the genus *Sodaliphilus* positively interacted with hydrogenotrophic methane production pathways [35]. *Sodaliphilus pleomorphus* is a type strain of the genus *Sodaliphilus*. Studies have indicated that its genome lacks genes encoding multiple glycolytic proteins and hydrogenases, in addition to its requirement for co-cultivation for better growth [34]. Therefore, we speculated that *Sodaliphilus* (MAG 45) in our study might provide formate and acetate to *Methanobrevibacter* (MAGs 1, 2, and 3). When *Ruminococcus* sp. was co-cultured with *Methanobrevibacter* sp., more acetate and H_2_ were produced [36]. Furthermore, the review also concluded that acetate, amino acids, and thiamine are required as growth factors by *Methanosphaera* and *Methanobrevibacter* [37]. Based on the dominant functions of MAGs in cluster 2, we speculated that *Ruminococcus_E bovis* (MAG 189), *CAG-603* (MAG 75), and *Sodaliphilus* (MAG 45) may provide growth factors for *Methanobrevibacter* (MAGs 1, 2, and 3) and *Methanosphaera* (MAG 4).

Several studies have investigated the potential role of rumen microbiota in the development of ketosis [18,38]. The results of these studies are consistent with our results regarding the changes in propionate concentration. *CAG-603* sp. belongs to the genus *Bovifimicola*. *Bovifimicola ammoniilytica* is a species of the genus *Bovifimicola*. It can assimilate NH_3_-N and synthesize Glu, producing acetate and propionate [39]. Generally, NH_3_-N is the end product of feed protein and is also the raw material used by rumen microbiota to produce MCP. A previous study described the nitrogen pathway of *Ruminococcus albus* and indicated that extracellular NH_3_-N is assimilated and then used for amino acid synthesis via the Glu–Gln cycle [40]. Bacteria, including *Ruminococcus_E bovis* (MAG 189) and *CAG-603* (MAG 75), had the highest numbers of genes involved in metabolism. *Ruminococcus_E bovis* belongs to the genus *Ruminococcus_E*, and the type strain JE7A12, has been isolated from the rumen contents of dairy cows [41]. Previous studies have shown that JE7A12 can ferment starch, d-glucose, d-galactose, d-fructose, maltose, and glycogen and produce acetate as the major metabolic product [41]. In the present study, we found several enriched amino acid metabolism pathways in *Ruminococcus_E bovis* (MAG 189). To explore the potential role of *Ruminococcus_E bovis* (MAG 189) in amino acid metabolism regulation, we conducted an in vitro experiment and added different amounts of *R. bovis* JE7A12. We found that Ala and other amino acid concentrations were increased by its addition, including Asp, Glu, Gly, Met, and Phe. Previous reviews have concluded that the genus *Ruminococcus_E* is a core member of the rumen microbiota [42]. Our study provided the possibility to regulate the amino acid composition of MCP to supply more suitable amino acids for dairy cows (Fig. 7). However, we also found Thr, Arg, Orn, and Glu concentrations were decreased after addition. We speculated that the change in amino acid composition may be attributed to the change in microbiota or the introduction of amino acid from *R. bovis* JE7A12. Considering the complex relations between microbiota composition and amino acid composition, further research is still needed to clarify the relationship between the microbial composition and the amino acid composition in the rumen. The rumen fluid used in the in vitro experiment of this study may also affect the colonization of the *R. bovis* JE7A12, as the probiotic colonization depends on individual responsiveness [43]. Both diet contribution and feed conditions, along with microbiota and process engineering issues, must be taken into consideration to replicate the digestive process and simulate the gastrointestinal tract. Hence, an animal experiment is necessary to evaluate in the future the effects of the addition of *R. bovis* JE7A12 on the rumen microbiome.

Our evidence indicated that the Ala concentration in the MCP was mostly correlated with serum amino acid and TAA concentrations and associated with serum Ala concentration. This suggests that Ala may play a key role in amino acid metabolism for postpartum dairy cows. Measurements of liver pyruvate carboxylase messenger RNA and utilization of Ala for glucose synthesis suggest a greater dependence on Ala by conjecture in the days following parturition [24]. Previous metabolomic analyses of the serum and milk levels of ketosis cows found that branched amino acid, Ala, Asp, and Glu metabolism and the relative amino acid concentrations of dairy cows were depressed [21,22]. The liver is the major organ in amino acid metabolism, and our results also showed that amino acid metabolism was depressed in the ketosis cows. For peripartum women, liver samples are difficult to obtain, while changes in serum metabolites during the peripartum period showed the branched chain amino acids as biomarkers of diabetes mellitus [44]. Trp also showed an association with postpartum maternal mood [45]. However, these amino acids were not changed in the ketosis dairy cows. This may be due to the different species. Next, we successfully established a ketogenic hepatocyte model and found that Ala supplementation was effective in depressing ketogenesis and enhancing glucose synthesis and the pentose phosphate pathway (Fig. 7). Furthermore, we also found that it could promote fatty acid degradation, which meant that hepatocytes could better cope with high NEFA mobilization and further support the energy demand of postpartum dairy cows. A study revealed that hepatic fatty acids’ β-oxidation is significantly increased in subclinical ketosis cows but markedly decreased in clinical ketosis cows, and hepatic fatty acid synthesis is significantly increased in the latter, which induces hepatic steatosis [16]. Therefore, we speculated that Ala might be the limiting amino acid for maintaining energy metabolism homeostasis in postpartum dairy cows. Generally, Lys and Met were the limiting amino acids for dairy cows to produce milk [17]. Amino acid nutrition in monogastric animals indicates that the limiting sequence of amino acid changes at different physiological stages [46]. Combining our results with those of previous studies, we suggested that Ala deficiency in the MCP may lead to the development of ketosis by decreasing glucose supplement and increasing muscle mobilization.

In this study, we provide compelling evidence suggesting that Ala deficiency, induced by the rumen microbiota, can be traced back to the absence of *Ruminococcus_E bovis*. This deficiency contributes to energy metabolic disorders by regulating the host’s amino acid metabolism, thereby reducing the availability of glucose in the liver. In cows, it compels the mobilization of body muscle protein and fat reserves, decreasing milk protein production. It should be noted that in this study, the effects of the addition of *R. bovis* JE7A12 and the impact of Ala on liver metabolism were verified through in vitro experiments. Future in vivo animal studies will enable a comprehensive evaluation of their impacts. Collectively, we identified microbiome-derived Ala as a key metabolite for maintaining energy metabolic homeostasis in dairy cows. This research paves the way for further investigations into potential therapeutic strategies for managing energy metabolism disorders in mammals, by targeting the microbiota–metabolite axis.

## Materials and Methods

### Study design

The animal feeding experiment was conducted at a commercial dairy farm in Datong, China (39°93′N, 113°18′E) from September 2022 to January 2023. Briefly, 211 healthy parturient Holstein cows were selected. Blood samples (10 ml) were obtained via the coccygeal vessels using blood collection needles and EDTA evacuated tubes (Beijing HuaXiaHengYuan Technology Co., Ltd., Beijing, China) when cows returned to their pen after morning milking. Immediately after collection, the BHBA concentrations were measured using a bovine-specific electronic BHBA handheld meter (Nova Vet, Nova Biomedical Corporation, MA, USA). The BHBA concentrations were measured 21 d before calving (hereafter referred to as day −21) and days 1, 3, 7, 14, and 21. Cows with BHBA levels ≥1.2 mmol/l in at least one blood sample were diagnosed with ketosis [47]. The fixed veterinarian director was employed to diagnose all diseases, and cows with diseases except for ketosis (56 cows) were excluded in this experiment. Then, 22 cows with ketosis and 41 healthy cows were saved because of complete blood and rumen fluid samples (6 time points). Subsequently, 13 ketosis dairy cows (KET) and 13 cows without the disease (CON) were selected for downstream analyses according to parity and milk production during the last parity. Detailed information is presented in Table S3. The individual BHBA concentrations at different time points are presented in Fig. S10. All cows in the KET group were diagnosed with subclinical ketosis (Fig. S10). On days −21 and 1, there were no cows in the KET group with BHBA concentrations ≥1.2 mmol/l. G*power (v 3.1.9.2) was used to calculate the power. The power was 1.0 based on effect size *f* = 0.25, *α* error probability = 0.05, total sample size = 156, number of groups = 2, number of measurements = 6, correlation among repeated measures = 0.5, and nonsphericity correction *ε* = 1 under analysis of variance (ANOVA) repeated measures, within-between interaction. The feed ingredients and nutrient composition of diets are presented in Table S4. The daily temperature and humidity were recorded by a COS-03 recorder (Shandong Renke Measurement and Control Technology Co., Ltd., Jinan, China). The average high and low temperatures were 10.8 and −5.8 °C, respectively. The average humidity was 65.9%.

### Milk sampling and measurements

Milk samples were collected on days 7, 14, and 21 from all of the dairy cows. The cows were milked 3 times daily (0700, 1300, and 1900 hours). DeLaval Rotary E500 (DeLaval Co., Ltd., Tianjin, China) was used to collect milk samples from a distributary facility. Milk samples (50 ml) were collected for 3 consecutive milkings (4:3:3) on days 7, 14, and 21 and submitted to the Shanxi Dairy Herd Improvement Center (Taiyuan, China) to assess the concentrations of protein, fat, lactose, and urea nitrogen using the mid-infrared method (Lactoscan, Entelbra). G*power (v 3.1.9.2) was used to calculate the power. The power was 0.99 based on effect size *f* = 0.25, *α* error probability = 0.05, total sample size = 78, number of groups = 2, number of measurements = 3, correlation among repeated measures = 0.5, and nonsphericity correction *ε* = 1 under ANOVA repeated measures, within-between interaction.

### Serum sampling and measurements

On days −21, 1, 3, 7, 14, and 21, the serum (10 ml) was obtained after BHBA measurement via centrifugation at 4,000 × *g* at 4 °C and then stored at −20 °C for biochemical analysis. The NEFA, glucose, triglyceride, total protein, albumin, globulin, BUN, and Cr levels were determined using a fully automatic biochemical analyzer (GF-D200, Gaomi Analytical Instrument Co. Ltd, Gaomi, China) combined with commercial kits (Nanjing Jiancheng Bioengineering Institute, Nanjing, China). The 3-MH concentration was determined using ultrahigh-performance liquid chromatography coupled to tandem mass spectrometry as described by a previous study [48]. For the amino acid composition analysis, serum pretreatment was conducted according to the method described by a previous study [49]. The analysis was performed using an EXion LC liquid chromatograph (AB Sciex Pte. Ltd., USA) coupled with an AB6500 Plus mass spectrometer (AB Sciex Pte. Ltd., USA).

### Rumen fermentation and MCP measurements

On days −21, 1, 3, 7, 14, and 21, rumen fluid samples of each animal were obtained via oral intubation (Wuhan Anscitech Animal Husbandry Technology Co., Ltd., Wuhan, China) and a 50-ml injector (Beijing HuaXiaHengYuan Technology Co., Ltd., Beijing, China) when the cows returned to their pen after morning milking. Details of the VFA, MCP, NH_3_-N, and amino acid compositions in the MCP analyses are provided in Text S1.

### Metagenomic sequencing

Rumen samples collected on days 3, 7, and 14 were selected for metagenomic analysis. The extracted total DNA was processed to construct metagenome shotgun sequencing libraries with insert sizes of approximately ~400 bp, using an Illumina TruSeq Nano DNA LT Library Preparation Kit (Illumina, USA). Each library was sequenced using an Illumina NovaSeq X Plus platform (Illumina, USA) with PE150 strategy at Personal Biotechnology Co., Ltd. (Shanghai, China). Details of data processing and statistical analysis are provided in Text S1.

### Metagenome assembly and binning

Only contigs with >300 bp were saved for analysis. Thereafter, MetaBinner (version 1.4.4) was used to bin contigs with default parameters [50]. All of the generated MAGs were taxonomically annotated using GTDB-Tk (version 2.3.0), which produced the standardized taxonomic labels that were used for the analysis in this study. Completeness and contamination were estimated using CheckM (version 1.1.6) [51], based on which these MAGs were classified as high-quality (complete >80%, contamination <5%) according to the previous criteria [52]. Details of data processing and statistical analysis are provided in Text S1.

### Liver tissue sampling and measurements

On day 21 after the collection of serum and rumen fluid samples, 10 dairy cows from each group were selected and their liver tissue samples were obtained via biopsy, as previously described [53]. The needle of the biopsy pistol collected approximately 15 mg of liver tissue biopsies. Samples were immediately stored in a 4% paraformaldehyde fixing solution (G-CLONE Biotechnology Co., Ltd, Beijing, China) or frozen in liquid nitrogen.

For liver PAS staining, metabolomics, and RNA-seq, details of these processes and statistical analyses are provided in Text S1.

### In vitro rumen fermentation adding *R. bovis*

The type strain (*R. bovis* JE7A12) was purchased from the American Type Culture Collection Center (TSD-225). The powder of this strain was activated according to the instructions and concentrated after growing to an appropriate concentration. After obtaining a suitable concentration of bacterial fluid, we prepared the fermentation system including 0.5 g of the substrate (diet of dairy cows), 25 ml of rumen fluid, and 50 ml of buffer. Before the fermentation system was ready, it had not been connected to the automated trace gas recording system (AGRS-III) yet. The same volume (75 μl) of bacterial fluid with different bacterial concentrations (0, 10^9^, 10^10^, and 10^11^ CFU/ml) was added into the 75-ml fermentation system to reach the target concentration (0, 10^6^, 10^7^, and 10^8^ CFU/ml *R. bovis* JE7A12). Each treatment included 5 bottles. After connecting AGRS and 48-h fermentation, the pH, fermentation parameters, and amino acid composition in MCP were analyzed as the in vivo study description.

### In vitro bovine hepatocyte model and treatment

Hepatocyte isolation and culture are described in Text S1. For the relatively high-glucose (HG) group, hepatocytes were maintained in Gibco Dulbecco’s modified Eagle’s medium (DMEM; Thermo Fisher Scientific Inc., USA) (4.5 g/l glucose) supplemented with 10% fetal bovine serum (Beijing Solarbio Science & Technology Co., Ltd., China) for 12 h. For the ketogenic hepatocyte (KETH) model, hepatocytes were treated with 1.2 mM NEFA mixture for 12 h in low-glucose DMEM (1 g/l glucose) (Thermo Fisher Scientific Inc., USA). Briefly, a stock fatty acid solution was prepared by diluting individual fatty acids in 0.1 mM NaOH. The composition of fatty acids in the NEFA mixture was oleic and palmitic (Sigma-Aldrich, St. Louis, MO, USA) at a ratio of 2:1 (oleic:palmitic) [54]. For the L-ALA and H-ALA groups, hepatocytes were treated with 1.2 mM NEFA mixture + 5 mM (L-ALA) or 10 mM Ala (H-ALA) (Beijing Solarbio Science & Technology Co., Ltd., China) for 12 h in low-glucose DMEM. After culturing, 1 ml of the supernatant was collected to analyze glucose, NEFA, and BHBA concentrations, and the hepatocytes were fixed with 4% paraformaldehyde for Oil Red O, PAS staining, and immunofluorescence. TRIzol (1 ml) was added into each well and mixed to remove the adherent cells for RNA-seq. Details of the analyses are provided in Text S1. The experiment was repeated 5 times.

### Statistical analysis

For the in vivo experiment, background information was compared between the 2 groups using a 2-sided Mann–Whitney *U* test. For milk composition, serum biochemical indices, and rumen fermentation parameters, the data were analyzed using the PROC MIXED procedure of SAS version 9.4 (SAS Institute Inc., Cary, NC, USA). Fixed effects included group (CON and KET), time (days −21, 1, 3, 7, 14, and 21), and interaction between group and time. The cow was included as a random effect. For liver-stained areas, the data were analyzed using a *t* test. The amino acid composition of the MCP was analyzed using the Wilcoxon test and corrected for multiple comparisons using the Bonferroni–Dunn method. A *P* value <0.05 was considered statistically significant. Spearman’s correlation was conducted using GraphPad Prism version 9.3 (GraphPad, MA, USA), and *P* < 0.05 was considered a significant correlation. Cytoscape (version 3.10.1) was used to visualize the connections.

For the in vitro fermentation experiment, total gas production, substrate degradability, and fermentation parameters were analyzed using ANOVA with Tukey’s test. Pearson’s linear and quadratic relationships were examined, and a *P* value <0.05 was considered statistically significant. Mfuzz clustering was used to show the changing trend of amino acid concentration among different groups (10 clusters).

For the in vitro hepatocyte experiment, hepatocyte supernatant indices, staining area, and MFI of key enzyme data were analyzed using ANOVA with Tukey’s test. A *P* value <0.05 was considered statistically significant.

## Data Availability

The raw data were deposited in the Sequence Read Archive database of the National Center for Biotechnology Information, and the project number is PRJNA976096 (Rumen microbiota transplantation and transition dairy cow microbiota regulation). The accession numbers of metagenomics are SRR29188398 to SRR29188472, SRR29188744, and SRR29188745. The accession numbers of RNA-seq are SRR30979202 to SRR30979211. The accession numbers of MAGs are SRR30984041 to SRR30984333.

## Supplementary Materials

Supplementary 1 Supplementary Materials and Methods Text S1 Figs. S1 to S10 Tables S1 to S4 References [55–74]

## References

[1] Gao S, Wang J. Maternal and infant microbiome: Next-generation indicators and targets for intergenerational health and nutrition care. Protein Cell. 2023;14(11):807–823. 37184065 10.1093/procel/pwad029 PMC10636639

[2] Baumgard LH, Collier RJ, Bauman DE. A 100-year review: Regulation of nutrient partitioning to support lactation. J Dairy Sci. 2017;100(12):10353–10366. 29153169 10.3168/jds.2017-13242

[3] Gu F, Zhu S, Tang Y, Liu X, Jia M, Malmuthuge N, Valencak TG, McFadden JW, Liu JX, Sun HZ. Gut microbiome is linked to functions of peripheral immune cells in transition cows during excessive lipolysis. Microbiome. 2023;11(1): Article 40. 10.1186/s40168-023-01492-3 PMC9983187 36869370

[4] Chirivi M, Rendon CJ, Myers MN, Prom CM, Roy S, Sen A, Lock AL, Contreras GA. Lipopolysaccharide induces lipolysis and insulin resistance in adipose tissue from dairy cows. J Dairy Sci. 2022;105(1):842–855. 34696909 10.3168/jds.2021-20855

[5] Lei L, Gao W, Loor JJ, Aboragah A, Fang Z, Du X, Zhang M, Song Y, Liu G, Li X. Reducing hepatic endoplasmic reticulum stress ameliorates the impairment in insulin signaling induced by high levels of β-hydroxybutyrate in bovine hepatocytes. J Dairy Sci. 2021;104(12):12845–12858. 34538494 10.3168/jds.2021-20611

[6] Ye W, Luo C, Huang J, Li C, Liu Z, Liu F. Gestational diabetes mellitus and adverse pregnancy outcomes: Systematic review and meta-analysis. BMJ. 2022;377: Article e067946. 35613728 10.1136/bmj-2021-067946 PMC9131781

[7] Diaz-Santana MV, O’Brien KM, Park Y-MM, Sandler DP, Weinberg CR. Persistence of risk for type 2 diabetes after gestational diabetes mellitus. Diabetes Care. 2022;45(4):864–870. 35104325 10.2337/dc21-1430 PMC9016728

[8] Proudfoot KL, Huzzey JM. A first time for everything: The influence of parity on the behavior of transition dairy cows. JDS Commun. 2022;3(6):467–471. 36465505 10.3168/jdsc.2022-0290 PMC9709596

[9] Kim JJ, Silver RK, Elue R, Adams MG, La Porte LM, Cai L, Kim JB, Gibbons RD. The experience of depression, anxiety, and mania among perinatal women. Arch Womens Ment Health. 2016;19(5):883–890. 27188618 10.1007/s00737-016-0632-6

[10] Vinet A, Drouilhet L, Bodin L, Mulsant P, Fabre S, Phocas F. Genetic control of multiple births in low ovulating mammalian species. Mamm Genome. 2012;23(11–12):727–740. 22872147 10.1007/s00335-012-9412-4

[11] Hudak SK, Overkamp D, Wagner R, Häring H-U, Heni M. Ketoacidosis in a non-diabetic woman who was fasting during lactation. Nutr J. 2015;14(1): Article 117. 26537818 10.1186/s12937-015-0076-2 PMC4634581

[12] Jaber JF, Standley M, Reddy R. Euglycemic diabetic ketoacidosis in pregnancy: A case report and review of current literature. Case Rep Crit Care. 2019;2019(1): Article 8769714. 31531246 10.1155/2019/8769714 PMC6721267

[13] Song Y, Wang K, Loor JJ, Jiang Q, Yang Y, Jiang S, Liu S, He J, Feng X, Du X, et al. β-Hydroxybutyrate inhibits apoptosis in bovine neutrophils through activating ERK1/2 and AKT signaling pathways. J Dairy Sci. 2022;105(4):3477–3489. 35151471 10.3168/jds.2021-21259

[14] Song Y, Li N, Gu J, Fu S, Peng Z, Zhao C, Zhang Y, Li X, Wang Z, Li X, et al. β-Hydroxybutyrate induces bovine hepatocyte apoptosis via an ROS-p38 signaling pathway. J Dairy Sci. 2016;99(11):9184–9198. 27756472 10.3168/jds.2016-11219

[15] Fan M, Song E, Zhang Y, Zhang P, Huang B, Yan K, Yang W, Chakrabarti S, Mahajan H, Yan S, et al. Metabolic dysfunction-associated steatohepatitis detected by neutrophilic crown-like structures in morbidly obese patients: A multicenter and clinicopathological study. Research. 2024;7: Article 0382. 38812532 10.34133/research.0382 PMC11134285

[16] Zhu Y, Liu G, Du X, Shi Z, Jin M, Sha X, Li X, Wang Z, Li X. Expression patterns of hepatic genes involved in lipid metabolism in cows with subclinical or clinical ketosis. J Dairy Sci. 2019;102(2):1725–1735. 30471902 10.3168/jds.2018-14965

[17] Schwab CG, Broderick GA. A 100-year review: Protein and amino acid nutrition in dairy cows. J Dairy Sci. 2017;100(12):10094–10112. 29153157 10.3168/jds.2017-13320

[18] Wang Q, Cui Y, Indugu N, Loor JJ, Jiang Q, Yu Z, Baker L, Pitta D, Deng Z, Xu C. Integrated meta-omics analyses reveal a role of ruminal microorganisms in ketone body accumulation and ketosis in lactating dairy cows. J Dairy Sci. 2023;106(7):4906–4917. 37296048 10.3168/jds.2022-22282

[19] Kong F, Wang S, Zhang Y, Li C, Dai D, Guo C, Wang Y, Cao Z, Yang H, Bi Y, et al. Rumen microbiome associates with postpartum ketosis development in dairy cows: A prospective nested case–control study. Microbiome. 2025;13(1): Article 69. 40057813 10.1186/s40168-025-02072-3 PMC11889851

[20] Bergman EN. Energy contributions of volatile fatty acids from the gastrointestinal tract in various species. Physiol Rev. 1990;70(2):567–590. 2181501 10.1152/physrev.1990.70.2.567

[21] Wang Y, Gao Y, Xia C, Zhang H, Qian W, Cao Y. Pathway analysis of plasma different metabolites for dairy cow ketosis. Ital J Anim Sci. 2016;15(3):545–551.

[22] Lisuzzo A, Laghi L, Faillace V, Zhu C, Contiero B, Morgante M, Mazzotta E, Gianesella M, Fiore E. Differences in the serum metabolome profile of dairy cows according to the BHB concentration revealed by proton nuclear magnetic resonance spectroscopy (^1^H-NMR). Sci Rep. 2022;12(1): Article 2525. 35169190 10.1038/s41598-022-06507-x PMC8847571

[23] Huang Y, Zhang B, Mauck J, Loor JJ, Wei B, Shen B, Wang Y, Zhao C, Zhu X, Wang J. Plasma and milk metabolomics profiles in dairy cows with subclinical and clinical ketosis. J Dairy Sci. 2024;107(8):6340–6357. 38608939 10.3168/jds.2023-24496

[24] Larsen M, Kristensen NB. Precursors for liver gluconeogenesis in periparturient dairy cows. Animal. 2013;7(10):1640–1650. 23823867 10.1017/S1751731113001171

[25] Li T-T, Chen X, Huo D, Arifuzzaman M, Qiao S, Jin W-B, Shi H, Li XV, Iliev ID, Artis D, et al. Microbiota metabolism of intestinal amino acids impacts host nutrient homeostasis and physiology. Cell Host Microbe. 2024;32(5):661–675. 38657606 10.1016/j.chom.2024.04.004 PMC11636940

[26] Lai M, Liu Y, Ronnett GV, Wu A, Cox BJ, Dai FF, Röst HL, Gunderson EP, Wheeler MB. Amino acid and lipid metabolism in post-gestational diabetes and progression to type 2 diabetes: A metabolic profiling study. PLOS Med. 2020;17(5): Article e1003112. 32433647 10.1371/journal.pmed.1003112 PMC7239388

[27] Wang X, Zhang Y, Zheng W, Wang J, Wang Y, Song W, Liang S, Guo C, Ma X, Li G. Dynamic changes and early predictive value of branched-chain amino acids in gestational diabetes mellitus during pregnancy. Front Endocrinol. 2022; Article 13. 10.3389/fendo.2022.1000296 PMC9614652 36313758

[28] Ma C, Sun Z, Zeng B, Huang S, Zhao J, Zhang Y, Su X, Xu J, Wei H, Zhang H. Cow-to-mouse fecal transplantations suggest intestinal microbiome as one cause of mastitis. Microbiome. 2018;6(1): Article 200. 30409169 10.1186/s40168-018-0578-1 PMC6225715

[29] Gu F, Zhu S, Hou J, Tang Y, Liu J-X, Xu Q, Sun H-Z. The hindgut microbiome contributes to host oxidative stress in postpartum dairy cows by affecting glutathione synthesis process. Microbiome. 2023;11(1): Article 87. 37087457 10.1186/s40168-023-01535-9 PMC10122372

[30] Koh A, Bäckhed F. From association to causality: The role of the gut microbiota and its functional products on host metabolism. Mol Cell. 2020;78(4):584–596. 32234490 10.1016/j.molcel.2020.03.005

[31] Miao G, Guo J, Zhang W, Lai P, Xu Y, Chen J, Zhang L, Zhou Z, Han Y, Chen G, et al. Remodeling intestinal microbiota alleviates severe combined hyperlipidemia-induced nonalcoholic steatohepatitis and atherosclerosis in LDLR^-/-^ hamsters. Research. 2024;7: Article 0363. 38694198 10.34133/research.0363 PMC11062505

[32] Kushitor SB, Owusu L, Kushitor MK. The prevalence and correlates of the double burden of malnutrition among women in Ghana. PLOS ONE. 2021;15(12): Article e0244362. 10.1371/journal.pone.0244362 PMC7769247 33370352

[33] Wu J-J, Zhu S, Tang Y-F, Gu F, Valencak TG, Liu J-X, Sun H-Z. Age- and microbiota-dependent cell stemness plasticity revealed by cattle cell landscape. Research. 2023;6: Article 0025. 37040481 10.34133/research.0025 PMC10076005

[34] Wylensek D, Hitch TCA, Riedel T, Afrizal A, Kumar N, Wortmann E, Liu T, Devendran S, Lesker TR, Hernández SB, et al. A collection of bacterial isolates from the pig intestine reveals functional and taxonomic diversity. Nat Commun. 2020;11(1): Article 6389. 33319778 10.1038/s41467-020-19929-w PMC7738495

[35] Park T, Cersosimo LM, Radloff W, Zanton GI, Li W. The rumen liquid metatranscriptome of post-weaned dairy calves differed by pre-weaning ruminal administration of differentially-enriched, rumen-derived inocula. Anim Microbiome. 2022;4(1): Article 4. 10.1186/s42523-021-00142-z PMC8728904 34983694

[36] Neumann L, Weigand E, Most E. Effect of methanol on methanogenesis and fermentation in the rumen simulation technique (RUSITEC). J Anim Physiol Anim Nutr. 1999;82(4):142–149.

[37] Khairunisa BH, Heryakusuma C, Ike K, Mukhopadhyay B, Susanti D. Evolving understanding of rumen methanogen ecophysiology. Front Microbiol. 2023;14: Article 1296008. 38029083 10.3389/fmicb.2023.1296008 PMC10658910

[38] Wang X, Li X, Zhao C, Hu P, Chen H, Liu Z, Liu G, Wang Z. Correlation between composition of the bacterial community and concentration of volatile fatty acids in the rumen during the transition period and ketosis in dairy cows. Appl Environ Microbiol. 2012;78(7):2386–2392. 22267666 10.1128/AEM.07545-11 PMC3302620

[39] Hitch TCA, Riedel T, Oren A, Overmann J, Lawley TD, Clavel T. Automated analysis of genomic sequences facilitates high-throughput and comprehensive description of bacteria. ISME Commun. 2021;1(1): Article 16. 10.1038/s43705-021-00017-z PMC9723785 36732617

[40] Kim JN, Henriksen ED, Cann I, Mackie RI. Nitrogen utilization and metabolism in *Ruminococcus albus* 8. Appl Environ Microbiol. 2014;80(10):3095–3102. 24610852 10.1128/AEM.00029-14 PMC4018901

[41] Gaffney J, Embree J, Gilmore S, Embree M. *Ruminococcus bovis* sp. Nov., a novel species of amylolytic *Ruminococcus* isolated from the rumen of a dairy cow. Int J Syst Evol Microbiol. 2021;71(8): Article 004924. 34379583 10.1099/ijsem.0.004924 PMC8513621

[42] Mizrahi I, Wallace RJ, Moraïs S. The rumen microbiome: Balancing food security and environmental impacts. Nat Rev Microbiol. 2021;19(9):553–566. 33981031 10.1038/s41579-021-00543-6

[43] Langella P, Chatel J-M. Risk assessment of probiotics use requires clinical parameters. Nat Rev Gastroenterol Hepatol. 2019;16(4):202–204. 30692658 10.1038/s41575-019-0111-4

[44] Chorell E, Hall UA, Gustavsson C, Berntorp K, Puhkala J, Luoto R, Olsson T, Holmäng A. Pregnancy to postpartum transition of serum metabolites in women with gestational diabetes. Metabolism. 2017;72:27–36. 28641781 10.1016/j.metabol.2016.12.018

[45] Bolton JL, Wiley MG, Ryan B, Truong S, Strait M, Baker DC, Yang NY, Ilkayeva O, O’Connell TM, Wroth SW, et al. Perinatal western-type diet and associated gestational weight gain alter postpartum maternal mood. Brain Behav. 2017;7(10): Article e00828. 29075574 10.1002/brb3.828 PMC5651398

[46] Wu G, Bazer FW, Burghardt RC, Johnson GA, Kim SW, Li XL, Satterfield MC, Spencer TE. Impacts of amino acid nutrition on pregnancy outcome in pigs: Mechanisms and implications for swine production. J Anim Sci. 2010;88(suppl_13):E195–E204. 19854987 10.2527/jas.2009-2446

[47] Gebreyesus G, Difford GF, Buitenhuis B, Lassen J, Noel SJ, Hojberg O, Plichta DR, Zhu Z, Poulsen NA, Sundekilde UK, et al. Predictive ability of host genetics and rumen microbiome for subclinical ketosis. J Dairy Sci. 2020;103(5):4557–4569. 32197852 10.3168/jds.2019-17824

[48] Sampsonidis I, Marinaki M, Pesiridou A, Gika H, Theodoridis G, Siachos N, Arsenos G, Kalogiannis S. Cost-effective simultaneous determination of τ- and π-methylhistidine in dairy bovine plasma from large cohort studies using hydrophilic interaction ultra-high performance liquid chromatography coupled to tandem mass spectrometry. Separations. 2023;10(2): Article 144.

[49] Toledo MZ, Nienow C, Luchini D, Arriola Apelo SI, Wiltbank MC. Quantification of bovine plasma amino acids via liquid chromatography–electrospray ionization-mass spectrometry: Comparison of underivatized and precolumn derivatized methods. JDS Commun. 2021;2(4):227–232. 36338448 10.3168/jdsc.2020-0060 PMC9623648

[50] Wang Z, Huang P, You R, Sun F, Zhu S. MetaBinner: A high-performance and stand-alone ensemble binning method to recover individual genomes from complex microbial communities. Genome Biol. 2023;24(1): Article 1. 10.1186/s13059-022-02832-6 PMC9817263 36609515

[51] Parks DH, Imelfort M, Skennerton CT, Hugenholtz P, Tyson GW. CheckM: Assessing the quality of microbial genomes recovered from isolates, single cells, and metagenomes. Genome Res. 2015;25(7):1043–1055. 25977477 10.1101/gr.186072.114 PMC4484387

[52] Bowers RM, Kyrpides NC, Stepanauskas R, Harmon-Smith M, Doud D, Reddy TBK, Schulz F, Jarett J, Rivers AR, Eloe-Fadrosh EA, et al. Minimum information about a single amplified genome (MISAG) and a metagenome-assembled genome (MIMAG) of bacteria and archaea. Nat Biotechnol. 2017;35(8):725–731. 28787424 10.1038/nbt.3893 PMC6436528

[53] Kong F, Wang F, Zhang Y, Wang S, Wang W, Li S. Repeated inoculation with rumen fluid accelerates the rumen bacterial transition with no benefit on production performance in postpartum Holstein dairy cows. J Anim Sci Biotechnol. 2024;15(1): Article 17. 10.1186/s40104-023-00963-9 PMC10838461 38310317

[54] Chen S, Che S, Li S, Ruan Z. The combined impact of decabromodiphenyl ether and high fat exposure on non-alcoholic fatty liver disease *in vivo* and *in vitro*. Toxicology. 2021;464: Article 153015. 34757160 10.1016/j.tox.2021.153015

[55] Kong F, Gao Y, Tang M, Fu T, Diao Q, Bi Y, Tu Y. Effects of dietary rumen–protected Lys levels on rumen fermentation and bacterial community composition in Holstein heifers. Appl Microbiol Biotechnol. 2020;104(15):6623–6634. 32519120 10.1007/s00253-020-10684-y

[56] Broderick GA, Kang JH. Automated simultaneous determination of ammonia and total amino acids in ruminal fluid and in vitro media. J Dairy Sci. 1980;63(1):64–75. 7372898 10.3168/jds.S0022-0302(80)82888-8

[57] Makkar H, Sharma O, Dawra R, Negi SS. Simple determination of microbial protein in rumen liquor. J Dairy Sci. 1982;65:2170–2173. 7153399 10.3168/jds.S0022-0302(82)82477-6

[58] Virág D, Király M, Drahos L, Édes AE, Gecse K, Bagdy G, Juhász G, Antal I, Klebovich I, Dalmadi Kiss B, et al. Development, validation and application of LC–MS/MS method for quantification of amino acids, kynurenine and serotonin in human plasma. J Pharm Biomed Anal. 2020;180: Article 113018. 31851908 10.1016/j.jpba.2019.113018

[59] Fuertig R, Ceci A, Camus SM, Bezard E, Luippold AH, Hengerer B. LC-MS/MS-based quantification of kynurenine metabolites, tryptophan, monoamines and neopterin in plasma, cerebrospinal fluid and brain. Bioanalysis. 2016;8(18):1903–1917. 27524289 10.4155/bio-2016-0111

[60] Kechin A, Boyarskikh U, Kel A, Filipenko M. cutPrimers: A new tool for accurate cutting of primers from reads of targeted next generation sequencing. J Comput Biol. 2017;24(11):1138–1143. 28715235 10.1089/cmb.2017.0096

[61] Chen S, Zhou Y, Chen Y, Gu J. fastp: An ultra-fast all-in-one FASTQ preprocessor. Bioinformatics. 2018;34(17):i884–i890. 30423086 10.1093/bioinformatics/bty560 PMC6129281

[62] Li H. Minimap2: Pairwise alignment for nucleotide sequences. Bioinformatics. 2018;34(18):3094–3100. 29750242 10.1093/bioinformatics/bty191 PMC6137996

[63] Menzel P, Ng KL, Krogh A. Fast and sensitive taxonomic classification for metagenomics with Kaiju. Nat Commun. 2016;7: Article 11257. 27071849 10.1038/ncomms11257 PMC4833860

[64] Li D, Liu CM, Luo R, Sadakane K, Lam TW. MEGAHIT: An ultra-fast single-node solution for large and complex metagenomics assembly via succinct *de Bruijn* graph. Bioinformatics. 2015;31(10):1674–1676. 25609793 10.1093/bioinformatics/btv033

[65] Steinegger M, Söding J. MMseqs2 enables sensitive protein sequence searching for the analysis of massive data sets. Nat Biotechnol. 2017;35(11):1026–1028. 29035372 10.1038/nbt.3988

[66] Hyatt D, Chen GL, Locascio PF, Land ML, Larimer FW, Hauser LJ. Prodigal: Prokaryotic gene recognition and translation initiation site identification. BMC Bioinformatics. 2010;11: Article 119. 20211023 10.1186/1471-2105-11-119 PMC2848648

[67] Liao Y, Smyth GK, Shi W. featureCounts: An efficient general purpose program for assigning sequence reads to genomic features. Bioinformatics. 2014;30(7):923–930. 24227677 10.1093/bioinformatics/btt656

[68] Olm MR, Brown CT, Brooks B, Banfield JF. dRep: A tool for fast and accurate genomic comparisons that enables improved genome recovery from metagenomes through de-replication. ISME J. 2017;11(12):2864–2868. 28742071 10.1038/ismej.2017.126 PMC5702732

[69] Ondov BD, Treangen TJ, Melsted P, Mallonee AB, Bergman NH, Koren S, Phillippy AM. Mash: Fast genome and metagenome distance estimation using MinHash. Genome Biol. 2016;17(1): Article 132. 27323842 10.1186/s13059-016-0997-x PMC4915045

[70] Asnicar F, Thomas AM, Beghini F, Mengoni C, Manara S, Manghi P, Zhu Q, Bolzan M, Cumbo F, May U, et al. Precise phylogenetic analysis of microbial isolates and genomes from metagenomes using PhyloPhlAn 3.0. Nat Commun. 2020;11(1): Article 2500. 32427907 10.1038/s41467-020-16366-7 PMC7237447

[71] Zhang C, Shao Q, Liu M, Wang X, Loor JJ, Jiang Q, Cuan S, Li X, Wang J, Li Y, et al. Liver fibrosis is a common pathological change in the liver of dairy cows with fatty liver. J Dairy Sci. 2023;104(4):2700–2715. 10.3168/jds.2022-22021 36823013

[72] Zhao Z, Yang H, Wang Z, Ai Z, Yang R, Wang Z, Wang T, Fu K, Zhang Y. Metabolomics analysis of the yolk of Zhijin white goose during the embryogenesis based on LC-MS/MS. PLOS ONE. 2024;19(2): Article e0297429. 38335168 10.1371/journal.pone.0297429 PMC10857567

[73] Shi C, Wang C, Zeng L, Peng Y, Li Y, Hao H, Zheng Y, Chen C, Chen H, Zhang J, et al. Triphenyl phosphate induced reproductive toxicity through the JNK signaling pathway in *Caenorhabditis elegans*. J Hazard Mater. 2023;446: Article 130643. 36586333 10.1016/j.jhazmat.2022.130643

[74] Zhang B, Li M, Yang W, Loor JJ, Liang Y, Wang S, Zhao Y, Guo H, Ma X, Yu L, et al. Mitochondrial dysfunction and endoplasmic reticulum stress in calf hepatocytes are associated with fatty acid-induced ORAI calcium release-activated calcium modulator 1 signaling. J Dairy Sci. 2020;103(12):11945–11956. 32981726 10.3168/jds.2020-18684

