# Supplementary material for: Alanine Derived from Ruminococcus_E bovis Alleviates Energy Metabolic Disorders during the Peripartum Period by Providing Glucogenic Precursors
Source: Research (Wash D C). 2025 Apr 25;8:0682. doi: 10.34133/research.0682 (PMC12022398; doi:10.34133/research.0682)
Supplement: Supplementary 1 — Supplementary Materials and Methods Text S1 Figs. S1 to S10 Tables S1 to S4 References [55–74] [file research.0682.f1.zip › Supplementary material.docx]

**Title**

Alanine Derived from *Ruminococcus_E Bovis* Alleviates Energy Metabolic Disorders during the Peripartum Period by Providing Glucogenic Precursors

**Authors**

Fanlin Kong ^1†^, Shuo Wang ^1†^, Yijia Zhang ^2^, Chen Li ^3^, Dongwen Dai ^4^, Yajing Wang ^1^, Zhijun Cao ^1^, Hongjian Yang ^1^, Wei Wang ^1^*, and Shengli Li ^1^*

**Affiliations**

^1^ State Key Laboratory of Animal Nutrition and Feeding, Department of Animal Nutrition and Feed Science, College of Animal Science and Technology, China Agricultural University, Beijing 100193, China

^2^ Laboratory of Animal Neurobiology, Department of Basic Veterinary Medicine, College of Veterinary Medicine, Nanjing Agricultural University, Nanjing 210095, China

^3^ Department of Animal Nutrition and Feed Science, College of Animal Science, Xinjiang Agricultural University, Urumqi 830052, China

^4^ Department of Animal Nutrition and Feed Science, College of Animal Science and Technology, Ningxia University, Yinchuan 750021, China

* Address correspondence to: Wei Wang; wei.wang@cau.edu.cn and Shengli Li; lishengli@cau.edu.cn

^†^ These authors contributed equally to this work

**SUPPLEMENTARY MATERIAL AND METHODS**

**VFA, MCP, NH_3_-N, and AA composition analyses**

The initial 50 mL rumen content was discarded to avoid saliva contamination and the subsequent 15 mL aliquot was stored in a storage tube at -20 ℃ to measure rumen fermentation parameters, and a 2 mL aliquot was stored in a storage tube at -80 ℃ for metagenome sequencing. The VFA compositions were determined using an Agilent G689N gas chromatograph and Agilent G7683 autosampler (Agilent Technologies, Inc., CA, USA) according to the previous description [55]. Samples were centrifuged at 4,000 × g for 10 min to obtain a clear supernatant. The supernatant was analyzed for rumen ammonia (NH_3_-N) using a phenol-hypochlorite assay [56] with a microplate reader (SpectraMax iD3, Molecular Devices Co., Ltd., Shanghai, China). For MCP, the rumen liquor was shaken in a magnetic stirrer for 45 sec to remove the microbes adsorbed on the feed particles. Then, it was centrifuged at 408 × g for 5 min to remove the remaining feed particles. Aliquots of 1.5 mL each were centrifuged at 22,000 × g for 20 min to obtain microbes. Then, supernatants were discarded and the deposition was then used to determine the AA composition of microbes. The same deposition obtained was used to determine MCP concentration. Briefly, the remains were suspended in 0.5 mL NaOH (0.25 mmol/L) and heated in a boiling water bath for 10 min. Contents then were centrifuged at 25,000 × g for 30 min to obtain the supernatants. A total of 25 μL supernatant with 75 μL deionized water was then added into a 10 mL centrifugal tube adding 5 mL Coomassie brilliant blue to color MCP (SpectraMax iD3, Molecular Devices Co., Ltd., Shanghai, China) [57].

For the AA composition of MCP, the LC-MS method was used to quantify the AA composition according to previous studies [58,59]. Briefly, the microbial deposition of rumen fluid was extracted in 600 μL of 10% formic acid in methanol-water (1:1, V:V) with two steel balls and mixed by the vortex mixer for 30 s. The mixture was then treated by a tissue grinder (MB-96, Meibi Instrument Co., Ltd., Jiaxing, China) at 55 Hz for 90 s and then centrifuged at 12,000 × g at 4 ℃ for 5 min. After diluting 10 times, 100 μL Trp-d3 was added to the 100 μL mixture and passed through a 0.22 μm filter membrane into the LC-MS bottle. The analysis was performed on EXion LC liquid chromatography (AB Sciex Pte. Ltd., U.S.A) coupled with AB6500 Plus mass spectrometer (AB Sciex Pte. Ltd., U.S.A).

**Metagenomics data processing and statistical analysis**

For metagenomics data processing, sequencing adapters were removed from raw reads using Cutadapt (v 1.2.1) [60]. Then, low-quality reads were trimmed using a sliding-window algorithm in fastp (v 0.23.2) [61]. Reads were aligned to the bovine genome using Minimap2 (v2.24-f1122) to remove host contamination [62]. Following this, taxonomical classifications of metagenomics sequencing reads from each sample were performed using Kaiju (v1.9.0) against a Genome Taxonomy Database-derived (GTDB, Release 214) (http://gtdb.ecogenomic.org/) [63]. Reads assigned to metazoans or viridiplantae were removed for downstream analysis. Megahit (v1.1.2) was used for the assembly of reads in each sample using the “--k-list 33,55,77,99,127 --min-contig-len 300” presetted parameters [64]. The generated contigs were pooled together and clustered using Mmseqs2 (v15) with “easy-linclust” mode, setting the sequence identity threshold to 0.95 and covering residues of the shorter contig to 90% [65]. The lowest common ancestor taxonomy of the non-redundant contigs was obtained by aligning them against the NCBI-nt database by mmseqs2 with “taxonomy” mode, and contigs assigned to Viridiplantae or Metazoa were dropped in the following analysis. Prodigal (v2.6.3) was used to predict the genes in the contigs [66]. CDS sequences of all samples were clustered using mmseqs2 with “easy cluster” mode, setting protein sequence identity threshold to 0.95 and covered residues of the shorter contig to 90%. Then, the reads were mapped onto the predicted gene sequences using Minimap2 and featureCounts to count the number of reads aligned to the gene sequences for each gene [67]. The high-quality reads from each sample were mapped onto the predicted gene sequences using Minimap2 with “-ax sr –sam-hit-only” and using featureCounts to count the number of reads aligned to gene sequences to obtain the Reads Count for each gene [67]. The functionality of the non-redundant genes was obtained by annotation using mmseqs2 with the “search” mode against the KEGG.

For metagenomics, the co-occurrence network was conducted and the features of the network were analyzed. The Chao1 index, observed species number, and bray-Curtis distance of microbial composition and function between the two groups at different time points were compared via the Wilcoxon test.

For metagenome-assembled genomes, dRep (3.4.2) was used for the dereplication of MAGs in two steps [68]. First, MAGs were divided into primary clusters using Mash [69] at a 90% Mash average ANI. Then, each primary cluster was used to form secondary clusters at the threshold of 99% ANI with at least 30% overlap between genomes to obtain non-redundant MAGs (nrMAG). The phylogenetic tree of the nrMAGs was built using PhyloPhlAn (v3.0) [70]. The tree was visualized using iTOL v6.9.1 (https://itol.embl.de/). Open Reading Frames (ORFs) were predicted using Prodigal (v2.6.3) [66]. The ORF catalog was functionally annotated with KEGG orthologous group IDs KO and level using online GhostKOALA with the KEGG GENES database of the “genus_prokaryotes” option. The gene number in each KEGG L3 pathway for individual samples was presented in an expression matrix, which was then used for clustering analysis based on JSD distance. The optimal clustering number was determined via the CH index.

**Liver metabolomics and RNA-Seq analyses**

For the paraffin-embedded formalin-fixed section, liver tissue was treated according to the previous study [71] and cut into 4-μm thick sections. Periodic Acid Schiff staining solution suit (Servicebio Technology Co., Ltd., Wuhan, China) was used to stain the liver sections. The CaseViewer (2.3) and Image Pro Plus (6.0) were used to quantify the PAS-stained areas. The total area stained positive for PAS was measured and related to the total area per liver sample.

For liver metabolomics, liver tissue was cut on dry ice into a 2 mL Eppendorf tube. Then, the tissue samples with 200 μL of H_2_O and five ceramic beads were homogenized and 800 μL methanol/acetonitrile (1:1, V: V) were added for metabolite extraction. The mixture was centrifuged for 20 min (14,000 g, 4℃), then the supernatant was injected. Analysis was performed using a UHPLC (Vanquish UHPLC, Thermo Fisher Scientific Inc, U.S.A) coupled to a Qrbitrap (Q Exactive HF-X, Thermo Fisher Scientific Inc, U.S.A). The separation, gradient elution, and instrument parameters were adopted according to the previous study [72].

For liver RNA-Seq, total RNA was isolated using the Trizol Reagent (Thermo Fisher Scientific Inc, U.S.A), after which the concentration, quality, and integrity were determined using a NanoDrop spectrophotometer (Thermo Fisher Scientific Inc, U.S.A). Three micrograms of RNA were used as input material for the RNA sample preparations and sequencing libraries were generated according to the description of Shi et al [73] The sequencing library was then sequenced on NovaSeq 6000 platform (Illumina).

The different metabolites were filtered via this threshold of Variable Importance in the Projection > 1 and *P* < 0.05, and different genes were filtered via *P* < 0.05. Statistical significance was calculated using DESeq2 with BH multiple comparisons. Significantly different metabolites or genes were then enriched in the KEGG pathway respectively, and the significantly different pathways were filtered via *P* < 0.05 based on BH adjustment. The Principal Component Analysis based on Z-score transformation was used to visualize the metabolome separation between groups. Finally, the significantly different metabolism pathways on KEGG L3 from both metabolomics and transcriptomics were enrolled in network analysis via the metabolism relationship.

**In vitro bovine** **hepatocyte model and treatment**

Isolation and Culture of Primary Hepatocytes were conducted by Zhang et al [74]. The growth medium was composed of Gibco Dulbecco's Modified Eagle Medium (DMEM) and 10% fetal bovine serum (FBS). The growth medium was replaced with a fresh medium every 24 h. Bovine hepatocyte was cultured in 6-well plates with 5 × 10^4^ cells/mL and then cultured for 12 h until 80% cell density. Hepatocytes were serum-free starved in Gibco DMEM basic medium for 6 h to subsequent treatments. After sample collection, glucose, NEFA, and BHBA concentrations in the supernatant were measured via commercial kits (Nanjing Jiancheng Bioengineering Institute, Nanjing, China). PAS and Oil red O staining solution suit (Servicebio Technology Co., Ltd., Wuhan, China) were used to stain the hepatocyte. The CaseViewer (2.3) and Image Pro Plus (6.0) were used to quantify the PAS and Oil red O-stained areas. The total area stained positive was measured and related to the total area per liver sample. For RNA-Seq, the RNA extraction and sequencing were conducted the same as liver tissue.

For hepatocyte RNA-Seq data processing, PCA analysis was used to show the distribution of samples. Statistical significance was calculated using DESeq2 with BH multiple comparisons. Significantly different genes (up-regulated or down-regulated) were then enriched in the KEGG pathway respectively, and the significantly different pathways were filtered via *P* < 0.05 based on BH *P*-value adjustment. The heatmap plot of differentially expressed genes are shown based on the Z-score. Cluster analysis of samples was conducted via complete and Euclidean.

Cell slides were fixed with 4% paraformaldehyde for 20 min, and penetrated with Permeabilize solution (G1204, Servicebio, Wuhan, China) for 20 min. Then, the cells were blocked with 3% normal goat serum for 30 min and incubated with antibodies (1:100) at 4 °C overnight, and specific signals were visualized with HRP secondary antibody (Servicebio, Wuhan, China). The cells were counterstained with 1 μg/mL DAPI (G1012, Servicebio, Wuhan, China) and visualized by a Nikon Eclipse C1 upright Microscopes (Nikon Imaging Japan Inc., Tokyo, Japan) and Pannoramic MIDI (3DHISTECH Ltd., Hungary). The CaseViewer (2.3) and Image Pro Plus (6.0) were used to quantify the MFI of key enzymes. The MFI is calculated by dividing the fluorescence intensity by the area.

**SUPPLEMENTARY FIGURES**


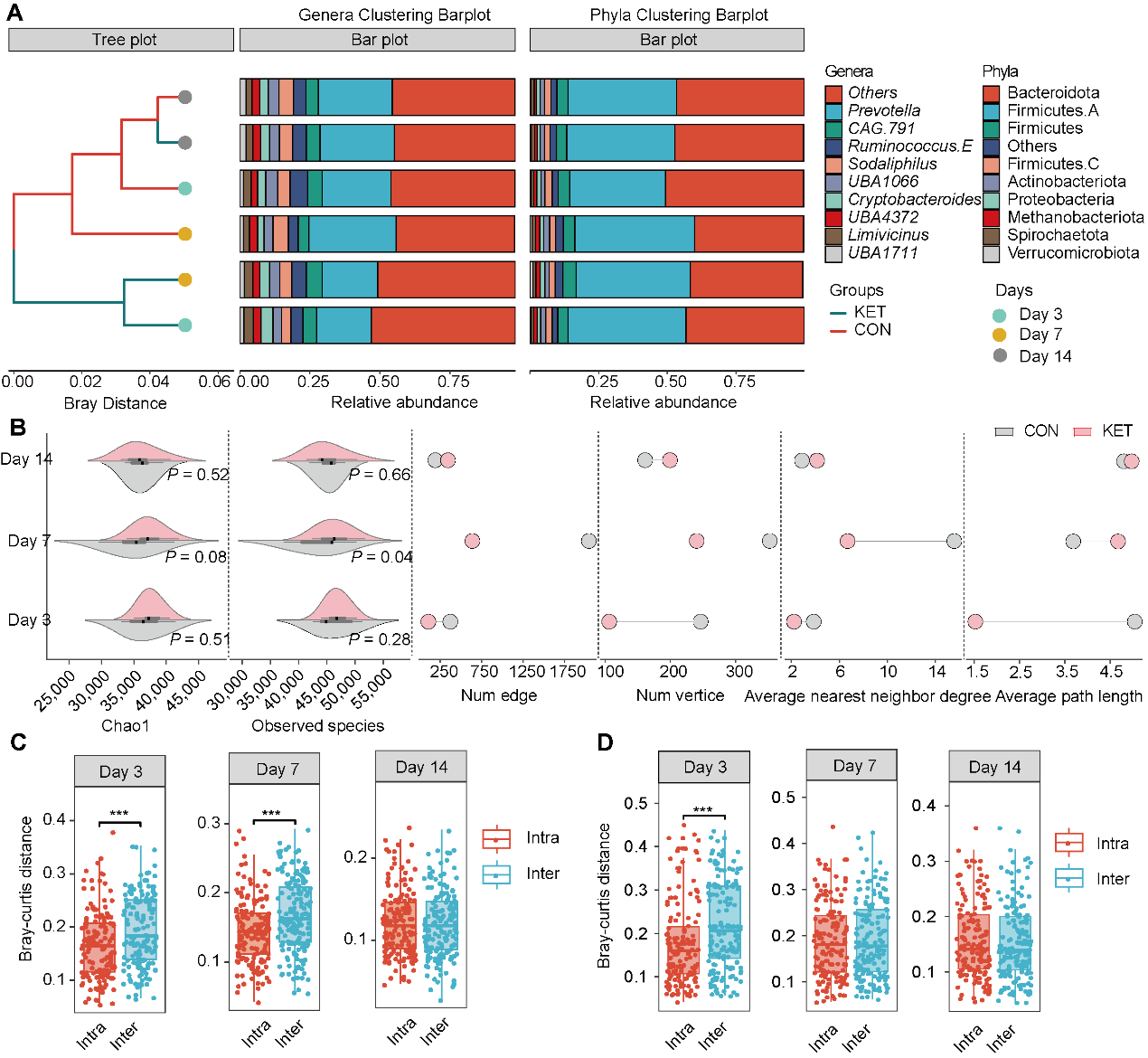


**Fig. S1.** Metagenome analysis for rumen microbiome composition and function changes of healthy (CON) and ketosis (KET) cows on days 3, 7, and 14 after calving. (A) the clustering tree was generated via Bray-Curtis on the general level. The top 10 taxa were presented. (B) variations in the alpha diversity indices and microbial network properties. Alpha indices including Chao1 and observed species were compared using a two-tailed Wilcoxon rank test. The mean value of the dataset was represented by the black line across the box plot. Network topology from rumen genera on days 3, 7, and 14. R^2^ > 0.6 or < - 0.6 were considered as cut-off values. (C, D) intra distance within each group on days 3, 7, and 14 and inter distance between two groups on different times were calculated based on genus (C) and function (D) composition using Anosim analysis. The boxplots show the mean (center line), quartiles (box limits), and max to min range (whiskers). Dots correspond to individual samples. *P* values were calculated using a two-tailed Wilcoxon rank test. * *P* < 0.05; ** *P* < 0.01; *** *P* < 0.001.


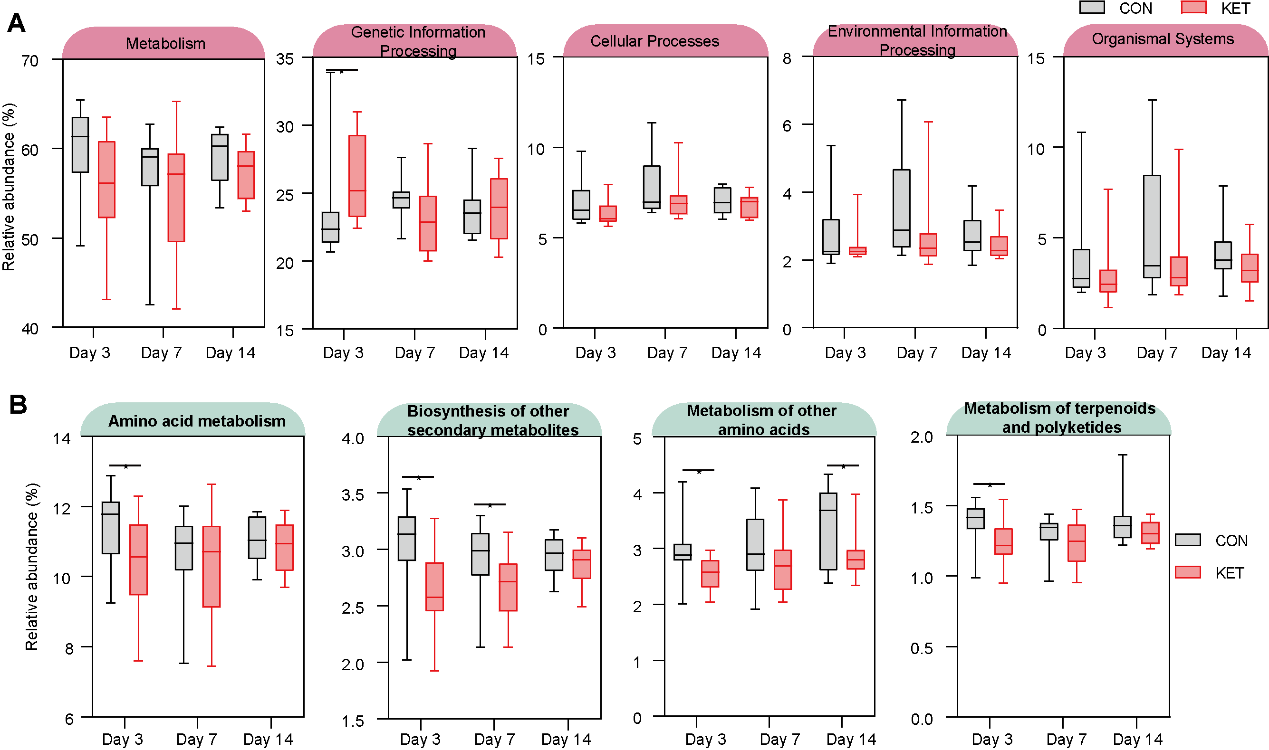


**Fig. S2.** KEGG Level 1 and Level 2 pathway relative abundances of the rumen microbiome in healthy (CON) and ketosis (KET) cows on days 3, 7, and 14 after calving. (A) KEGG Level 1 relative abundance. The boxplots show the mean (center line), quartiles (box limits), and max to min range (whiskers). *P* values were calculated using a two-tailed Wilcoxon rank test and corrected for multiple comparisons using the Bonferroni-Dunn method. * *P* < 0.05; ** *P* < 0.01; *** *P* < 0.001. (B) KEGG Level 2 metabolism pathway relative abundances. Only significantly different pathways were presented. The statistical analyses were conducted as Level 1.


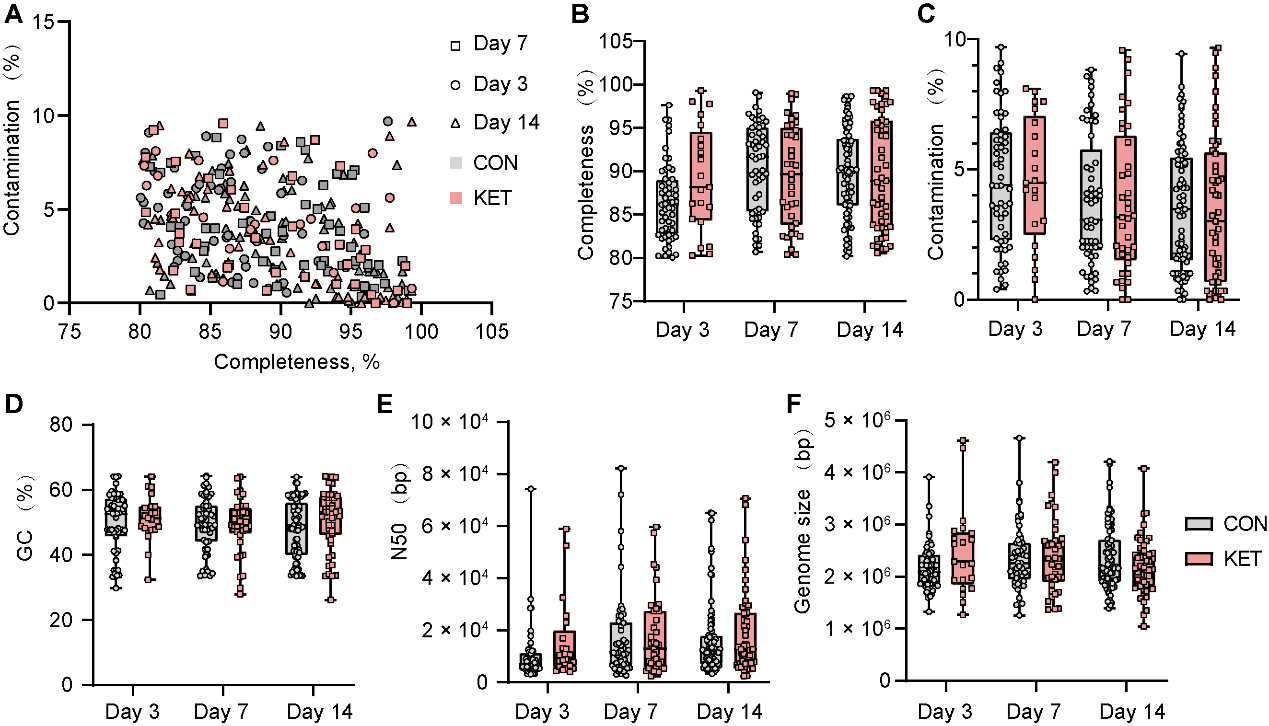


**Fig. S3.** Features of metagenome-assembled genomes (MAG) from the rumen microbiome of healthy (CON) and ketosis (KET) cows at different time points. (A) threshold of complete > 90%, and contamination < 5% was used to filter high-quality MAGs. (B-F) completeness (B), contamination (C), GC content (D), N50 (E), and genome size (F) of high-quality MAGs. The boxplots show the mean (center line), quartiles (box limits), and max to min range (whiskers). Dots correspond to individual MAG. *P* values were calculated using a two-tailed Wilcoxon rank test. * *P* < 0.05; ** *P* < 0.01; *** *P* < 0.001.


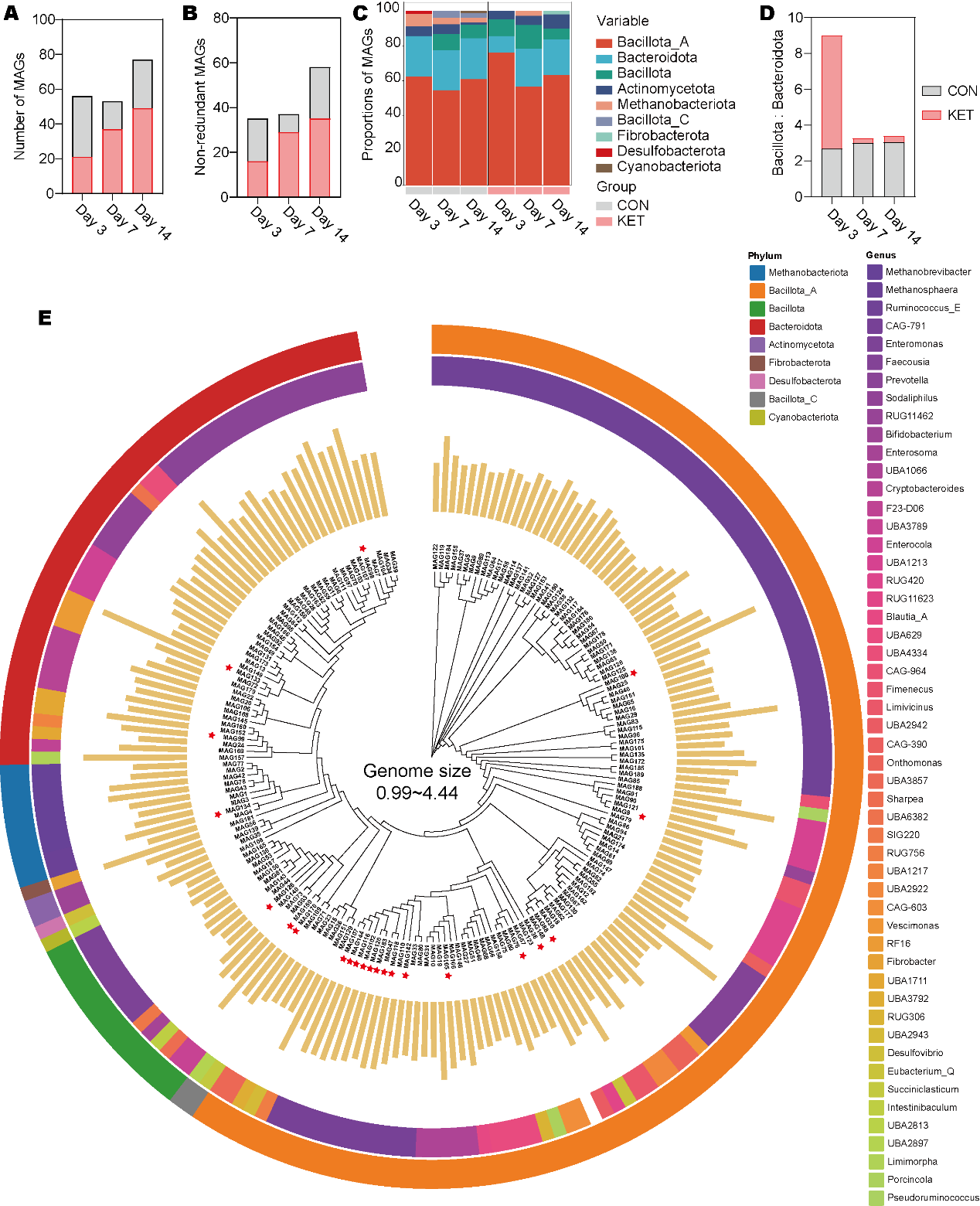


**Fig. S4.** Longitudinal changes of number and genus composition, and phylogeny of metagenome-assembled genomes (MAG) from the rumen of healthy (CON) and ketosis (KET) cows during the postpartum period. (A) number of high-quality (complete > 90%, contamination < 5%) MAGs. (B) number of non-redundant MAG (nrMAG). We dereplicated the high-quality MAGs at an ANI threshold of 99%to obtain the nrMAGs. (C) the phylum composition of MAGs. (D) the ratio of Bacillota: Bacteroidota of MAGs. (E) taxonomy tree of the species represented by 190 nrMAGs. Outermost bands with different colors show the phyla of all lineages. Next bands show the genus label. The bar chart indicates the genome size of each MAG. The asterisk indicates the MAG with no taxonomic assignment at the species level.


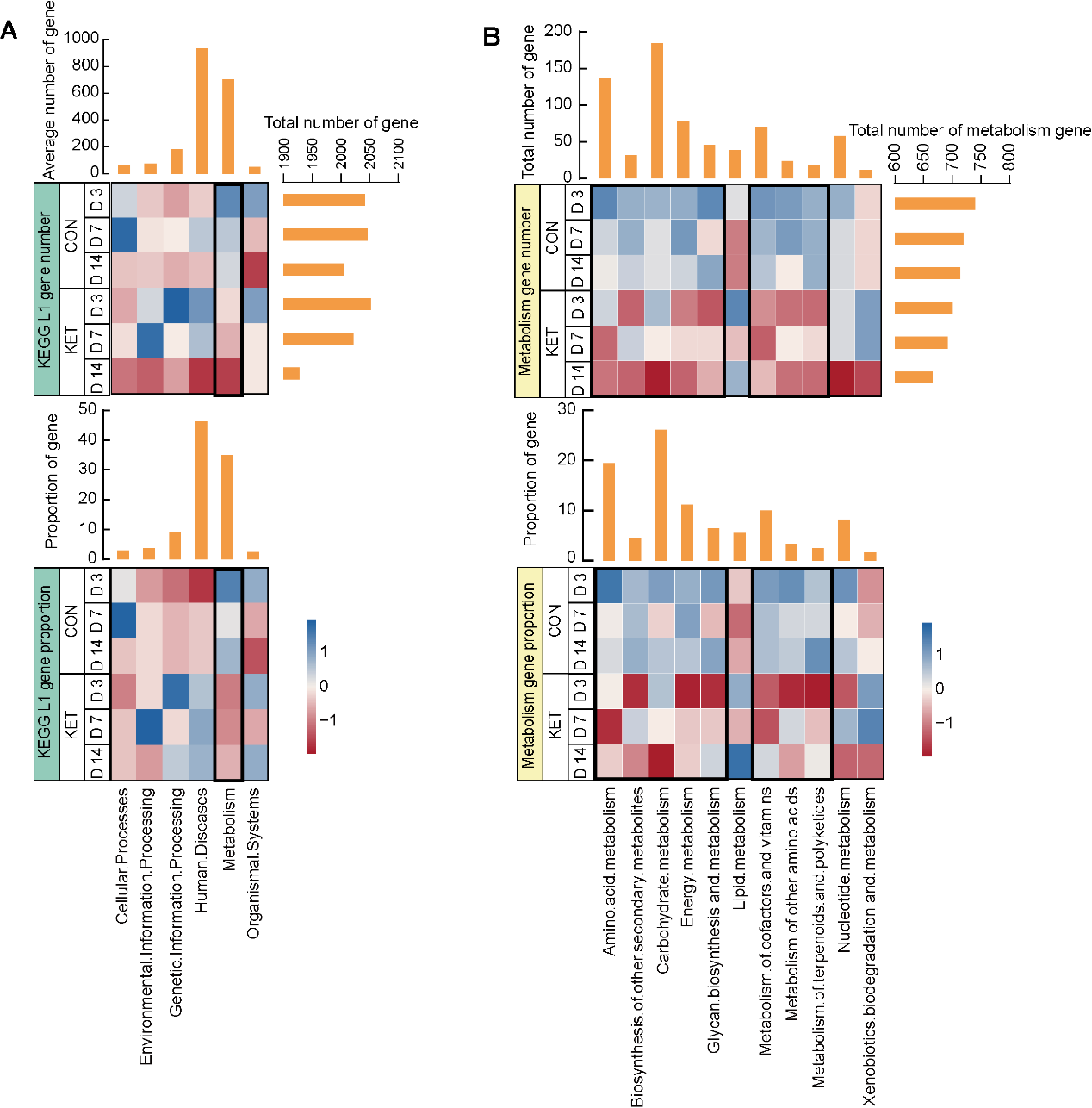


**Fig. S5.** Longitudinal changes of total functional gene number and proportion of all metagenome-assembled genomes (MAG) from the rumen microbiome of healthy (CON) and ketosis (KET) cows at different time points. (A) the proportion and number of gene groups in the KEGG level 1 pathway. The heatmap plots show the gene number and proportion of every KEGG level 1 pathway in each MAG. The data was presented via Z-score normalization. The vertical bar charts show the average proportion and number of genes of all MAGs. The horizontal bar charts show the total gene number of the CON and KET groups on different days. (B) the proportion and number of gene grouping in the sub-pathways of the METABOLISM pathway.


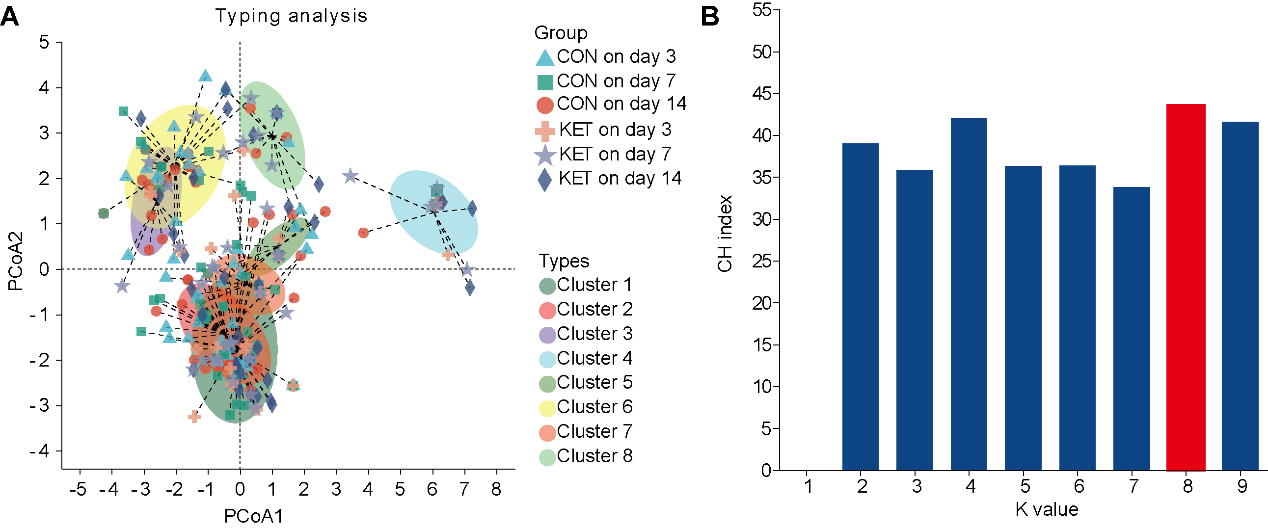


**Fig. S6.** The grouping of MAGs by KEGG level 3 pathway compositions and the CH index of individual K value of clustering analysis of MAGs. (A) the principal coordinates analysis (PCoA) plot of MAGs in different clusters. (B) the corresponding CH index of individual cluster number (K value).


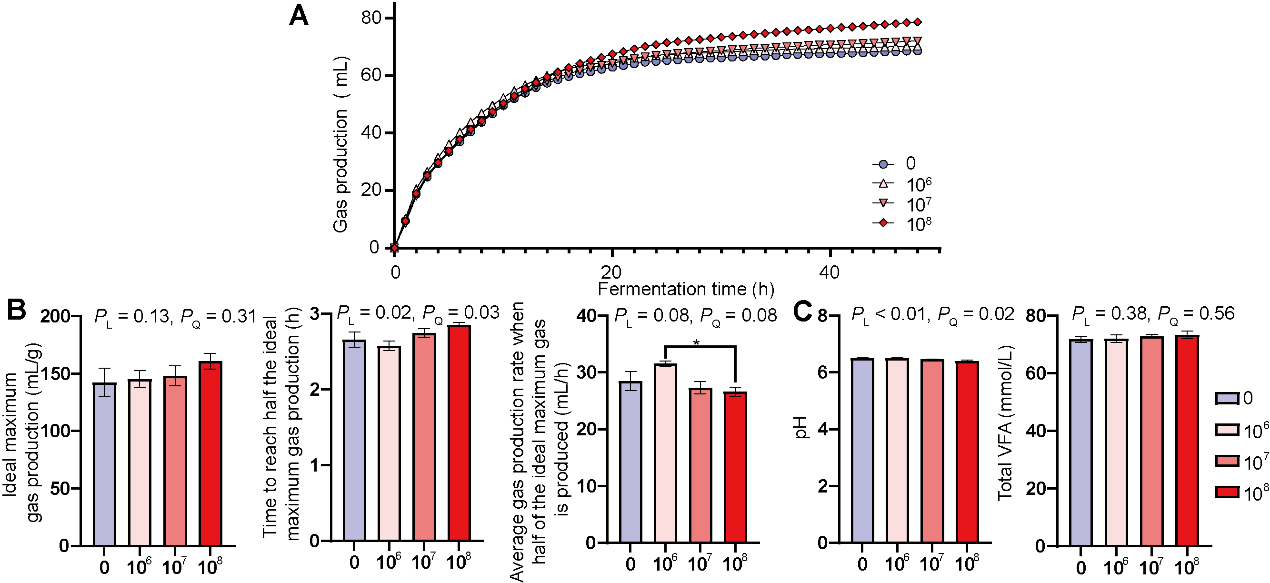


**Fig. S7.** Effects of in vitro *Ruminococcus bovis* supplementation on gas production and fermentation paramteters at 48 h. (A) the cumulative gas production per hour shown after 48 h fermentation. (B) gas production parameters. Statistical significance was calculated using one-way ANOVA with Tukey multiple comparisons. Linear and quadratic relationships were examined and *P*-values were presented as *P*_L_ and *P*_Q_. * *P* < 0.05; ** *P* < 0.01; *** *P* < 0.001. (C) fermentation parameters. Statistical significance was calculated using one-way ANOVA with Tukey multiple comparisons. Linear and quadratic relationships were examined and *P*-values were presented as *P*_L_ and *P*_Q_. * *P* < 0.05; ** *P* < 0.01; *** *P* < 0.001.


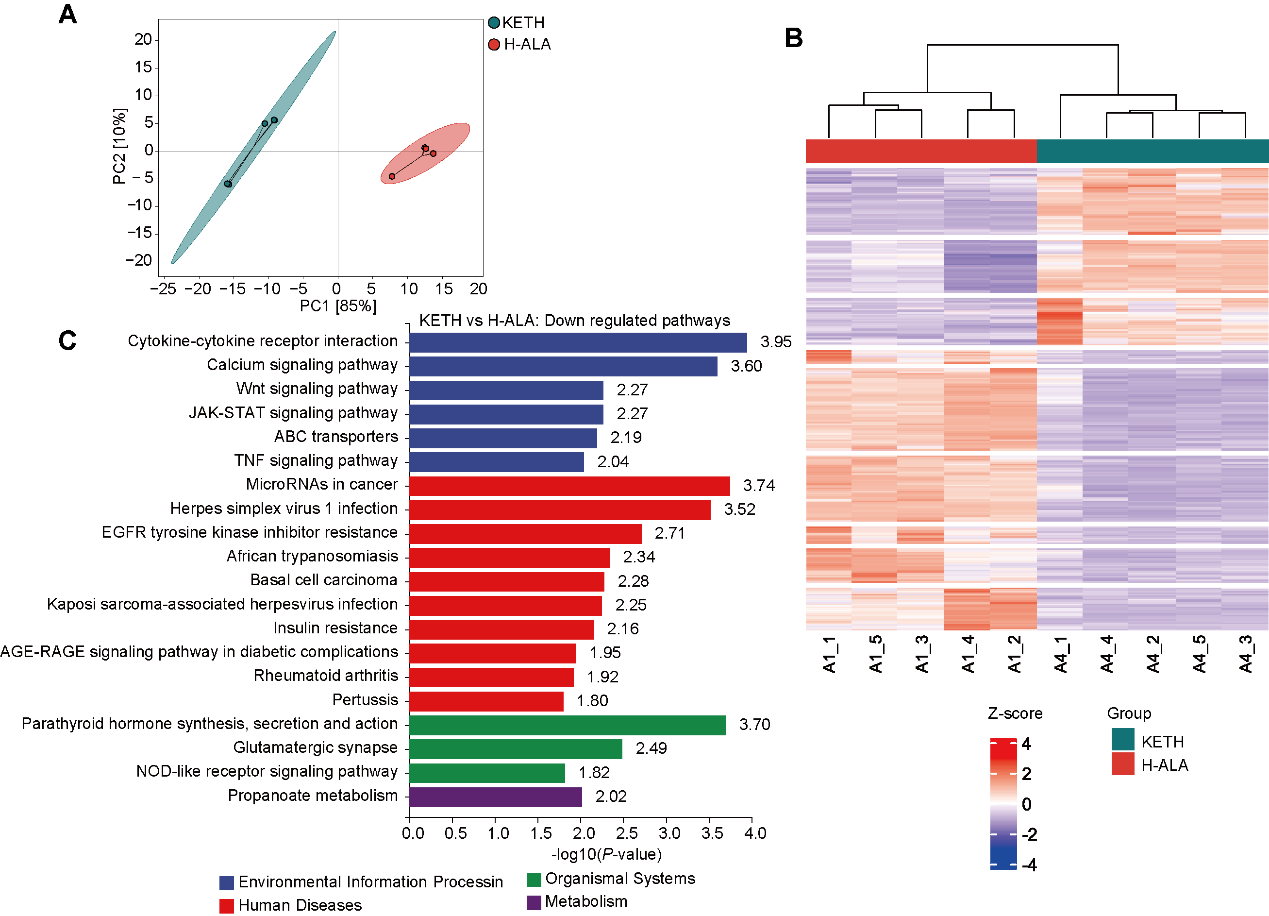


**Fig. S8.** RNA-Seq analysis for hepatic cells maintained in DMEM/low glucose with 1.2 mm NEFA (KETH) and 10 mm alanine additional supplementation (H-ALA). (A) PCA plot shows the distribution of samples in different groups. (B) the heatmap plot of differentially expressed genes (DEG). The data was normalized by Z-score. Cluster analysis of samples was conducted via complete and Euclidean. (C) significantly downregulated pathways of DEG in the H-ALA group compared with the KETH group. The top 20 *P*-values of pathways were presented.


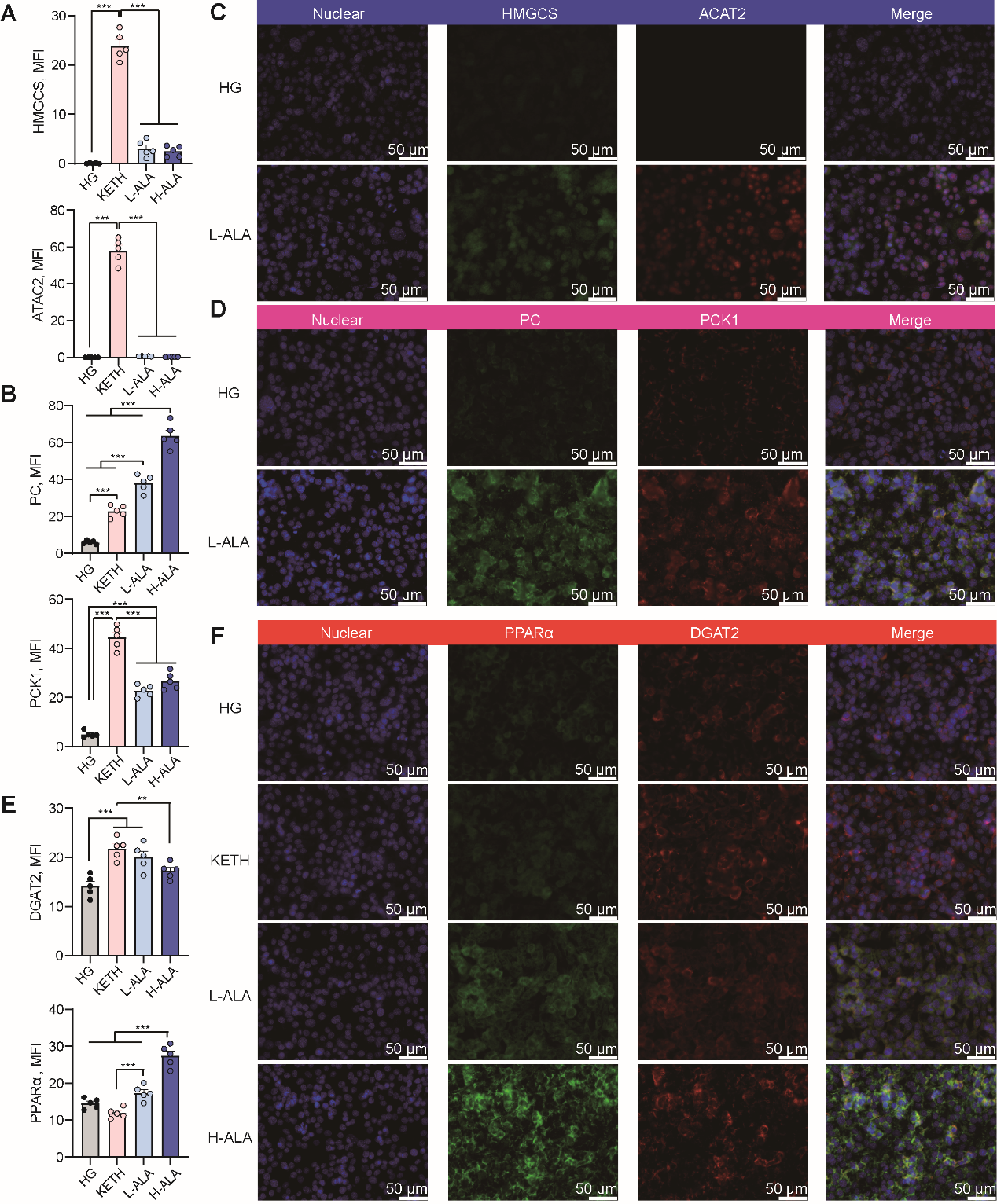


**Fig. S9.** Immunofluorescence for key enzymes of gluconeogenesis, ketogenesis, and lipid metabolism. Hepatic cells in the HG group were maintained in DMEM/Gibco (4.5 g/L). Hepatic cells in the KETH group were maintained in DMEM/low glucose (1 g/L) and supplied with 1.2 Mm NEFA for 12 h. Hepatic cells in L-ALA and H-ALA groups were also maintained in DMEM/low glucose with 1.2 Mm NEFA and then supplied with 5 or 10 Mm alanine for 12 h. (A, B, E) Quantification of mean fluorescence intensity (MFI) of HMGCS, ACAT2, PC, PCK1, PPARα and DGAT2. (C) the key enzymes of ketogenesis including HMGCS and ACAT2. (D) the key enzymes of gluconeogenesis including PC and PCK1. (F) the key enzymes of fatty acid degradation (PPARα) and triglyceride (DGAT2). Statistical significance was calculated using one-way ANOVA with Tukey multiple comparisons. * *P* < 0.05; ** *P* < 0.01; *** *P* < 0.001.


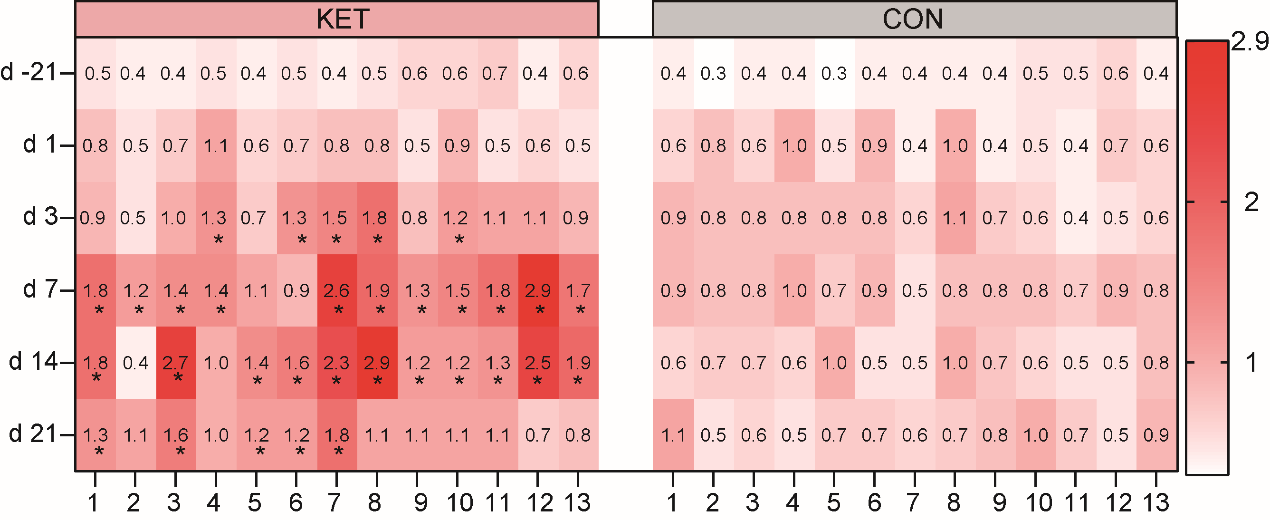


**Fig. S10. The distribution of BHBA concentrations of cows in the healthy (CON) and ketosis (KET) groups during the transition period.** The asterisk indicated that the BHBA concentration is between 1.2 and 3 mmol/L.

**SUPPLEMENTARY TABLES**

Table S1 (The ANI between the two MAGs) and S2 (Taxonomic attribution of MAGs) were uploaded in Excel format.

**Table S3.** Cohort description

| Items | CON | KET | *P*-value |
| --- | --- | --- | --- |
| Birth weight (kg) | 37.83 ± 3.49 | 39.11 ± 3.91 | 0.49 |
| Milk production during the last parity (ton) | 9.33 ± 1.40 | 10.83 ±1.57 | 0.16 |
| Actual calving date – predicted date (day) | 0.33 ± 1.97 | -0.15 ± 2.34 | 0.75 |
| Age (years) | 4.33 ± 0.75 | 4.68 ± 0.99 | 0.18 |
| Parity | 2.67 ± 0.78 | 2.85 ± 1.14 | 0.89 |

^a)^ Statistical significance was assessed using a two-sided Mann–Whitney U-test.

^b)^ Data are expressed as mean ± SEM.

**Table S4.** The feed ingredients and nutrient composition of diets

| Items | Prepartum | Postpartum |
| --- | --- | --- |
| Ingredient, g/kg (Dry matter basis) |  |  |
| Corn silage | 258 | 232 |
| Oat hay | 145 | 53 |
| Wheat straw | 359 |  |
| Alfalfa silage |  | 22 |
| Alfalfa hay |  | 136 |
| Soybean hulls |  | 44 |
| Steam flaked corn |  | 27 |
| Soybean meal | 21 | 69 |
| Distillers dried grains with solubles | 57 | 80 |
| Cottonseed meal | 85 |  |
| Corn germ meal | 57 |  |
| Corn |  | 186 |
| Soypass |  | 71 |
| Rumen protected fat |  | 9 |
| Urea | 5 | 3 |
| Molasses |  | 34 |
| [Choline](javascript:;) |  | 1 |
| MgO |  | 1 |
| NaHCO^3^ |  | 7 |
| Fat power |  | 3 |
| Premixes^1^ | 14 | 23 |
| Nutrient composition, % (air-dry basis) |  |  |
| Primary moisture^2^ | 52.23 | 49.98 |
| Dry matter | 93.58 | 93.63 |
| Crude protein | 15.35 | 17.21 |
| Acid detergent fiber | 23.53 | 20.99 |
| Neutral detergent fiber | 37.31 | 32.25 |
| Calcium | 1.18 | 1.07 |
| Phosphorus | 0.59 | 0.54 |
| Ash content | 9.29 | 9.52 |
| Fat | 3.13 | 3.63 |
| Starch | 15.86 | 22.61 |

^1^ Mix (Cargill feed Co., Ltd., Yichun City, China) administered during the prepartum period contained 420 KIU vitamin A/kg, 126 KIU vitamin D3/kg, 4,200 mg vitamin E/kg, 470 mg Cu/kg, 1,800 mg Zn/kg, 1,800 mg Mn/kg, 24 mg I/kg, 21 mg Se/kg, 24 mg Co/kg. Mix (Cargill feed Co., Ltd., Yichun City, China) administered during the postpartum period contained 170 KIU vitamin A/kg, 56 KIU vitamin D3/kg, 1,600 mg vitamin E/kg, 460 mg Cu/kg, 1,800 mg Zn/kg, 1,600 mg Mn/kg, 28 mg I/kg, 14 mg Se/kg, 28 mg of Co/kg.

^2^ Primary moisture was measured and calculated based on fresh sample weight. Primary moisture = (fresh sample weight – air-dry sample weight)/fresh sample weight.
